# Learning Causal Biological Networks With the Principle of Mendelian Randomization

**DOI:** 10.3389/fgene.2019.00460

**Published:** 2019-05-21

**Authors:** Md. Bahadur Badsha, Audrey Qiuyan Fu

**Affiliations:** Department of Statistical Science, Center for Modeling Complex Interactions, Institute for Bioinformatics and Evolutionary Studies, University of Idaho, Moscow, ID, United States

**Keywords:** causal inference, graphical models, biological networks, bioinformatics, Mendelian randomization

## Abstract

Although large amounts of genomic data are available, it remains a challenge to reliably infer causal (i. e., regulatory) relationships among molecular phenotypes (such as gene expression), especially when multiple phenotypes are involved. We extend the interpretation of the Principle of Mendelian randomization (PMR) and present MRPC, a novel machine learning algorithm that incorporates the PMR in the PC algorithm, a classical algorithm for learning causal graphs in computer science. MRPC learns a causal biological network efficiently and robustly from integrating individual-level genotype and molecular phenotype data, in which directed edges indicate causal directions. We demonstrate through simulation that MRPC outperforms several popular general-purpose network inference methods and PMR-based methods. We apply MRPC to distinguish direct and indirect targets among multiple genes associated with expression quantitative trait loci. Our method is implemented in the R package MRPC, available on CRAN (https://cran.r-project.org/web/packages/MRPC/index.html).

## Introduction

**Figure 1:**
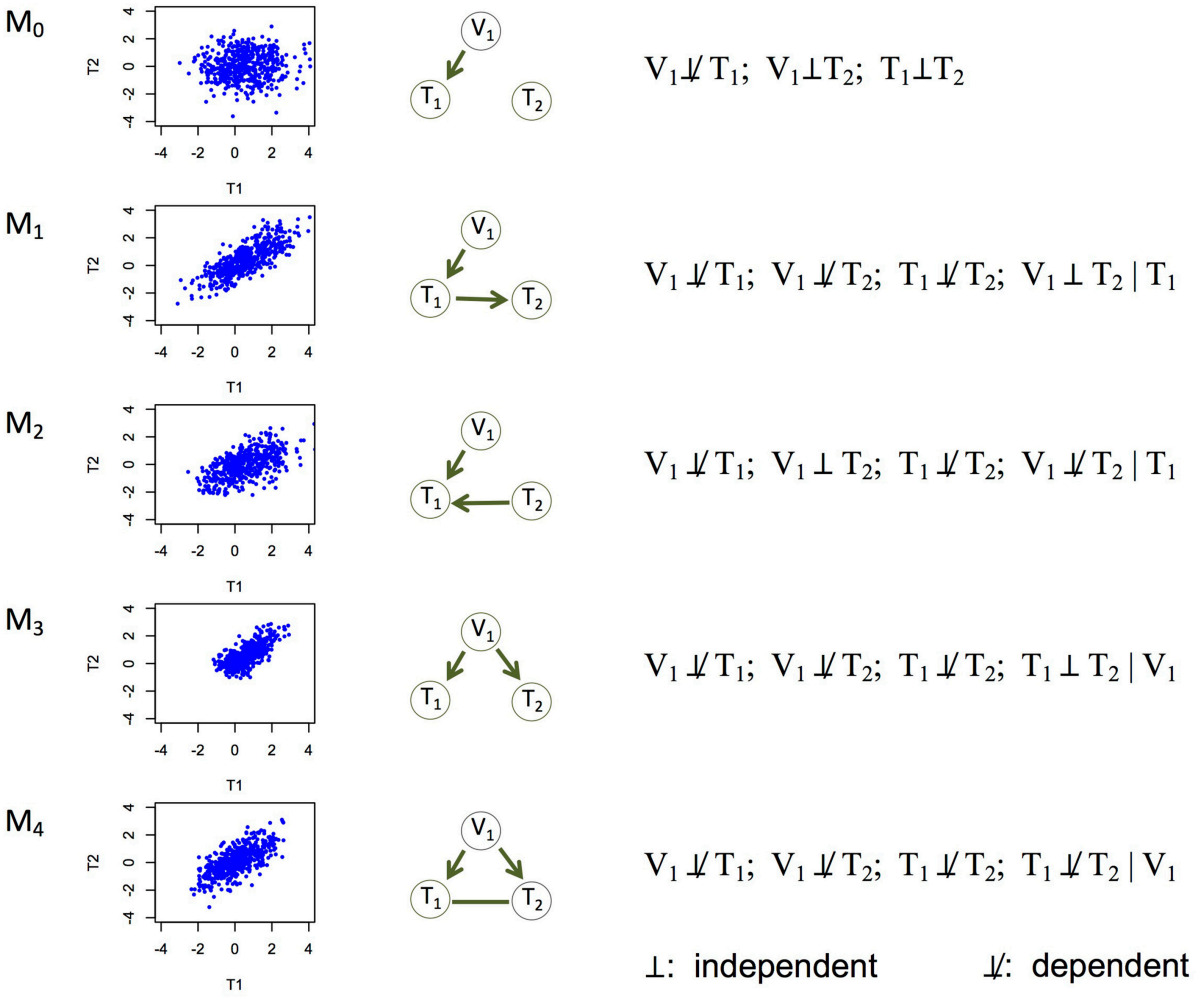
Five basic causal relationships under the principle of Mendelian randomization. Each topology involves three nodes: a genetic variant (V_1_), and two molecular phenotypes (T_1_ and T_2_). Directed edges indicate direction of causality, and undirected edges indicate that the direction is undetermined (or equivalently, both directions are equally likely). For each topology (or model), a scatterplot between the two phenotypes is generated using simulated data, the topology is shown, and the marginal and conditional dependence relationships are given. M_0_ is the null model where T_1_ and T_2_ are marginally independent, and therefore the scatterplot does not show correlation. All the other models show scatterplots with similar levels of correlation. M_1_ is the canonical causal model.

Experiments (e.g., temporal transcription or protein expression assays, gene knockouts or knockdowns) have been conducted to understand the causal relationships among genes (Segal et al., 2003; Housden et al., 2013), or between an expression Quantitative Trait Locus (eQTL) and its direct and indirect target genes (Cheung and Spielman, 2009). However, it remains a challenge to learn causality directly from genomic data. It is even harder to learn (i.e., infer) a causal network of multiple genes, which may represent which genes regulate which other genes (Hill et al., 2016; Ahmed et al., 2018). We address this problem in this paper. Correlation (or association) is often used as a proxy of a potentially causal relationship, but similar levels of correlation can arise from different causal mechanisms (Models 1–4 in Figure 1). For example, between two genes with correlated expression levels, it is plausible that one gene regulates the other gene (Models 1 and 2 in Figure 1); it is also plausible that they do not regulate each other directly, but both are regulated by a common genetic variant (Model 3 in Figure 1).

Correlation between the expression, or any molecular phenotype, of two genes is symmetrical—we cannot infer which of the two genes is the regulator and which the target. However, if a genetic variant (e.g., an eQTL) is significantly associated with the expression of one of the two genes, then we may assign a directed edge from the variant to the gene, as it is reasonable to assume that the genotype causes changes in the phenotype (expression), not the other way around. This additional, directed edge breaks the symmetry between the two genes, and makes it possible to infer the causal direction between them (e.g., compare Models 1 and 2 in Figure 1). This is the rationale behind the Principle of Mendelian Randomization (PMR). The randomization principle in experimental design (e.g., clinical trials) is critical in establishing causality: only when subjects are randomly assigned to different exposures is it possible to draw causal connections between exposure and outcome. By definition, the PMR is a randomization principle under which the alleles of a genetic variant are assumed to be randomly assigned to individuals in a population, analogous to a natural perturbation experiment and therefore achieving the goal of randomization (Davey Smith and Hemani, 2014). The above definition of the PMR implies three assumptions: (i) alleles of a genetic variant (or equivalently, genotypes) are causal to the associated phenotypes; (ii) the genetic variant is not associated with confounding variables that are also correlated with phenotypes; and (iii) the causal relationship cannot be explained by other variables. The PMR has been widely used in epidemiology studies, where genetic variants are used as instrumental variables to facilitate the estimate of causal effect between a mediator (or exposure, such as gene expression) and an outcome (e.g., a disease phenotype Davey Smith and Hemani, 2014). It received increasing attention in genetics in recent years (Millstein et al., 2009; Stojnic et al., 2012; Zhu et al., 2012; Gutierrez-Arcelus et al., 2013; Neto et al., 2013; Zhang et al., 2013; Huang et al., 2015; Oren et al., 2015; Franzén et al., 2016; Hill et al., 2016; Connor and Price, 2017; Wang and Michoel, 2017; Yang et al., 2017).

Large consortia, such as the Genetic European Variation in Disease (GEUVADIS) consortium (Lappalainen et al., 2013) and subsequently the Genotype-Tissue Expression (GTEx) consortium (The GTEx Consortium, 2017), have established the widespread genetic variation (i.e., eQTLs) in human genome that may regulate gene expression, making PMR-based methods increasingly relevant and important for understanding interactions among genes. Furthermore, Genome-Wide Association Studies (GWASs) have identified a large number of genetic variants that are potentially causal to diseases (MacArthur et al., 2017). Understanding the roles of these GWAS-significant variants is key to understanding the mechanisms underlying diseases. Interestingly, likely half of the GWAS-significant genetic variants are eQTLs (Nicolae et al., 2010). As it becomes more common nowadays to collect gene expression data in disease studies (Zhang et al., 2013; Franzén et al., 2016), studying eQTLs (which may also be GWAS-significant SNPs) and their associated genes provides a powerful approach for a deeper understanding of complex traits and diseases. An important application of causal network inference is then dissecting the relationships among multiple target genes of the same eQTL.

However, existing methods adopting the PMR, such as the mediation-based methods (Millstein et al., 2009; Wang and Michoel, 2017) and the Mendelian Randomization (MR) methods (Hemani et al., 2017; see Discussion on the relationships between these two classes of methods), are not directly applicable to inference of a causal network of gene expression. This is because these methods typically focus on the graph of V_1_ → T_1_ → T_2_ (i.e., Model 1 in Figure 1), where V_1_ is the genetic variant, T_1_ may represent gene expression, and T_2_ a clinical trait. This graph, considered the canonical causal model by existing PMR-based methods, is sensible when T_2_ is a potential outcome of T_1_. However, when we examine relationships among gene expression or other molecular phenotypes, it is usually not known beforehand which of T_1_ and T_2_ is more likely to be the outcome of the other, and Model 1 alone does not have the flexibility of examining additional possibilities. As a result, these methods are limited in the causal relationships they can recover. In this paper, we generalize the interpretation of the PMR to account for a variety of causal relationships, which enables us to infer more complex networks, although not at a high dimension yet.

**Figure 2:**
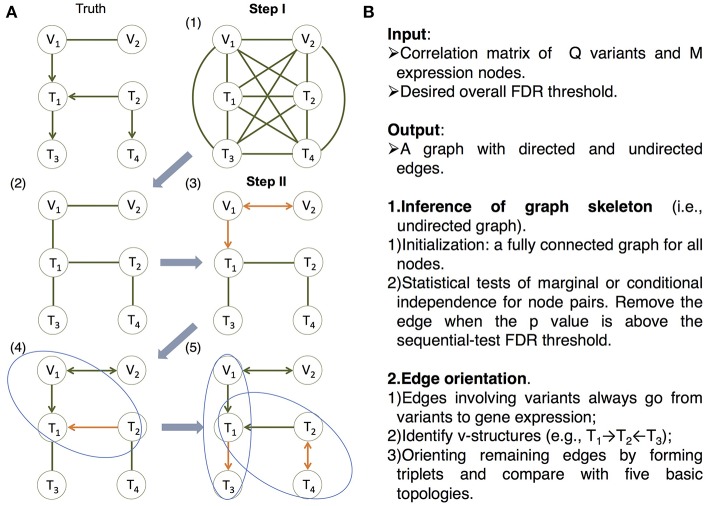
The MRPC algorithm. The MRPC algorithm consists of two steps. In Step I, it starts with a fully connected graph shown in (1), and learns a graph skeleton shown in (2), whose edges are present in the final graph but are undirected. In Step II, it orients the edges in the skeleton in the following order: edges involving at least one genetic variant (3), edges in a v-structure (if v-structures exist) (4), and remaining edges, for which MRPC iteratively forms a triplet and checks which of the five basic models under the PMR is consistent with the triplet (5). If none of the basic models matches the triplet, the edge is left unoriented (shown as bidirected). **(A)** An example illustrating the algorithm. **(B)** The pseudocode of the algorithm. See details in Figure S1 and an example for Step II in Figure S2.

On the other hand, in machine learning, a class of algorithms, such as those based on the classic PC algorithm (named after its developers Peter Spirtes and Clark Glymour; Spirtes et al., 2000; Tsamardinos et al., 2006; Scutari, 2010; Kalisch et al., 2012; Colombo and Maathuis, 2014), have been developed in over a decade to efficiently learn causal graphs for a large number of nodes. These algorithms typically consist of two main steps (Figure 2): (i) *inferring the graph skeleton* through a series of statistical independence tests. The graph skeleton is the same as the final graph except that the edges are undirected; and (ii) *determining the direction of the edges* in the skeleton. Variants of the original PC algorithm have been developed to reduce the impact of the ordering of the nodes on inference (e.g., the R package pcalg Kalisch et al., 2012; Colombo and Maathuis, 2014), or to reduce the number of statistical tests needed for inferring the skeleton (e.g., the R package bnlearn Tsamardinos et al., 2006; Scutari, 2010).

Here we develop a new method, namely MRPC, which incorporates the PMR into PC algorithms and goes beyond the canonical causal model. MRPC learns a causal graph where the nodes are genetic variants and molecular phenotypes (such as gene expression), and where the edges between nodes are undirected or directed, with the direction indicating causality. Crucially, by combining the PMR with machine learning, our method is efficient and accurate. Our extended interpretation of the PMR can be thought of as a way of introducing useful constraints in graph learning and effectively reducing the search space of topologies.

## Methods

### An Extended Interpretation of the Principle of Mendelian Randomization (PMR)

Consistent with other PMR-based methods, we represent the causal network among variables in terms of a set of marginal and conditional dependencies. For example, Model 1 (V_1_ → T_1_ → T_2_), the canonical causal model, can be represented mathematically as follows:

V_1_ and T_1_ are marginally dependent: V_1_⊥ T_1_;
V_1_ and T_2_ are marginally dependent: V_1_⊥ T_2_;
T_1_ and T_2_ are marginally dependent: T_1_⊥ T_2_;
V_1_ and T_2_ are conditionally independent given T_1_: V_1_⊥T_2_ | T_1_.

Defining causality through these dependencies means that if we can establish the statistical dependencies, then we can make causal statements.

The above model is under the standard interpretation of the PMR. Causal Inference Test (cit; Millstein et al., 2009, 2016) and Fast Inference of Networks from Directed Regulations (findr; Wang and Michoel, 2017), two existing PMR-based methods for example, both focus on testing this model. This model, however, is rather limited. Here we extend the interpretation of the PMR to consider five causal relationships in a triplet of a genetic variant and two phenotypes, including the canonical causal model (Figure 1). Under the assumptions that genotype influences phenotype and not the other way around and that confounding variables are absent, these five models are mutually exclusive and encompass all possibilities, with Model 0 being the null model where the two phenotype nodes are not related, and the other four models being non-null models.

Each of the other possible causal relationships also corresponds to a distinct set of marginal and conditional dependencies (Figure 1). Among them, Model 2 (V_1_ → T_1_←T_2_) defines a v-structure where both edges point to the same node. This model is suitable when no genetic variant is available for T_2_ in the data. This model is important to account for because although gene regulation by genetic variants is widespread, whether an eQTL is identified for a gene depends on the tissue type, sample size, and so on (The GTEx Consortium, 2017). Model 3 (V_1_ → T_1_ and V_1_ → T_2_) captures the scenario where T_1_ and T_2_ are not directly related, but both regulated by V_1_. The current interpretation of the PMR in other methods typically rejects these two models in search of the canonical causal model (Model 1). However, under our interpretation of the PMR, Models 2 and 3 describe alternative regulatory mechanisms between two genes, and therefore should also be allowed when constructing the network of molecular phenotypes. Model 4 (V_1_ → T_1_; V_1_ → T_2_; T_1_-T_2_) refers to the case where the two phenotypes T_1_ and T_2_ have additional dependence (represented by the undirected edge) on top of that induced by the sharing genetic variant. We consider undirected and bidirected edges to be equivalent for simplicity, in that an undirected edge can be thought of as representing two equally likely directions: T_1_ → T_2_ and T_1_←T_2_. It is plausible that a hidden variable regulates both T_1_ and T_2_, or affects the triplet in other ways. We describe how to identify and account for potentially confounding variables in section Accounting for Confounding Variables.

### MRPC, a Novel Causal Network Learning Algorithm

Our method, namely MRPC, is a novel causal network inference method for individual-level genomic data (Figure 2; Figures S1, S2). This method analyzes a data matrix with each row being an individual, and each column a genetic variant or a molecular phenotype. Similar to PC algorithms, our method also consists of two main steps. The first step of learning the graph skeleton is similar to that of PC algorithms: MRPC performs a series of statistical tests for marginal or conditional dependence in node pairs. If the dependence is not statistically significant, then the edge between the two nodes is removed. Unlike existing PC algorithms, though, MRPC implements an online control of the False Discovery Rate (FDR), which is explained in detail in the next section.

We incorporated the PMR in the second step of edge orientation (Figure 2; Figure S2). In this step, we deal with scenarios where the edge direction is more easily determined, before moving onto difficult situations. Specifically, MRPC first identifies edges involving the genetic variants and orient these edges to point to the molecular phenotype. Next, MRPC looks for three nodes that form a potential v-structure (e.g., Model 2 in Figure 1, or among three molecular phenotypes, T_1_ → T_2_←T_3_). The skeleton of a v-structure may be represented as X–Y–Z, where each node may be a genetic variant or gene expression. Four directed graphs are possible: (i) X → Y←Z; (ii) X → Y → Z; (iii) X←Y←Z; and (iv) X←Y → Z. Graphs (ii) through (iv) all represent conditional independence between X and Z given Y, and are therefore indistinguishable (they are termed Markov equivalent graphs; Richardson, 1997). Among these four possibilities, only the v-structure in graph (i) is uniquely determined. MRPC conducts additional conditional independence tests between X and Z given Y, if no such test has been performed in the first step. Among the remaining edges, MRPC iteratively finds node triplets with only one undirected edge. It examines the results from the independence tests from the first step to identify which of the five basic topologies is consistent with the test results for this triplet.

In MRPC, we use the standard Fisher's z transformation for Pearson correlation in all the marginal (Fisher, 1915) tests and for the partial correlation in all the conditional tests. Consider testing conditional (Fisher, 1924) independence between variables *x* and *y* conditioned on a set *S* of other variables. From the correlation matrix, one may estimate the partial correlations using an iterative approach (Kalisch et al., 2012). Then application of Fisher's z transformation gives the test statistic

$$\begin{array}{l} T=\frac{\sqrt{n-|S|-3}}{2}\log\left(\frac{1+{\hat{r}}_{x,y|S}}{{1-\hat{r}}_{x,y|S}}\right), \end{array}$$

which follows N(0,1) under the null hypothesis of conditional independence. In the expression above, *n* is the sample size, and |*S*| the number of variables in the set *S*, and ${\hat{r}}_{x,y|S}$ the estimated partial correlation. If the set *S* is empty, then we are testing marginal independence.

### Sequential FDR Control

Existing network inference algorithms (such as those implemented in R packages pcalg and bnlearn) control the type I error rate for each individual statistical test, but not the Family-Wise Error Rate (FWER) or the FDR, as most methods controlling both the FWER and FDR require the knowledge of the total number of tests, which is not known in advance in graph learning. Lack of correction for multiple comparison often leads to too many false edges in the inferred graph, especially when the graph is large (see our simulation results below).

We implemented in MRPC the Levels based on Number of Discoveries (LOND) method for controlling the FDR in an online manner (Javanmard and Montanari, 2015), as we generally do not know the number of tests beforehand in learning the causal graph. The LOND method estimates the expected FDR conditioned on the number of tests performed so far and the number of rejections from these tests. Specifically, consider a sequence of null hypotheses (marginal or conditional independence between two molecular phenotypes) *H*(*m*) = *H*_1_, *H*_2_, *H*_3_, …, *H*_*m*_, with corresponding *p*-values *p*(*m*) = *p*_1_, *p*_2_, *p*_3_, …, *p*_*m*_. The LOND algorithm aims to determine a sequence of significance level α_*i*_, such that the decision for the *i*th test is

$$R_{i}=\{\begin{array}{ccc} 1, & if\;p_{i}\;\leq \;\alpha_{i} & \left(\text{reject }H_{i}\right)\\ 0, & if\;p_{i}\;>\;\alpha_{i} & \left(\text{accept }H_{i}\right) \end{array}\;.$$

The number of rejections over *m* tests is then

$$\begin{array}{l} D_{\left(m\right)}=\sum_{i=\;1}^{m}R_{i}. \end{array}$$

For the overall FDR to be δ, the significance level α_*i*_ is set to be

$$\begin{array}{l} \alpha_{i}=\delta_{i}\left[D_{\left(i-1\right)}+1\;\right], \end{array}$$

where the FDR for the *i*th test is

$$\begin{array}{l} \delta_{i}=\;\frac{C}{i^{a}}, \end{array}$$

such that

$$\begin{array}{l} \sum_{i=1}^{\infty }\delta_{i}=\;\delta , \end{array}$$

for integer *a* > 1 and a constant *c*. We choose a nonnegative sequence $\delta_{i},\;such\;that\;\overset{\infty }{\underset{i=1}{\sum }}\delta_{i}=FDR.\;$ The default value for *a* is set to 2 in MRPC. At an FDR of 0.05 and *a* = 2, we have

$$\begin{array}{l} \overset{\infty }{\underset{i=1}{\sum }}\delta_{i}=\overset{\infty }{\underset{i=1}{\sum }}\frac{c}{i^{2}}=c\overset{\infty }{\underset{i=1}{\sum }}\frac{1}{i^{2}}=\frac{c\pi^{2}}{6}=0.05. \end{array}$$

Then

$$\begin{array}{l} c=\frac{6\times 0.05}{\pi^{2}}=0.0304. \end{array}$$

**Figure 3:**
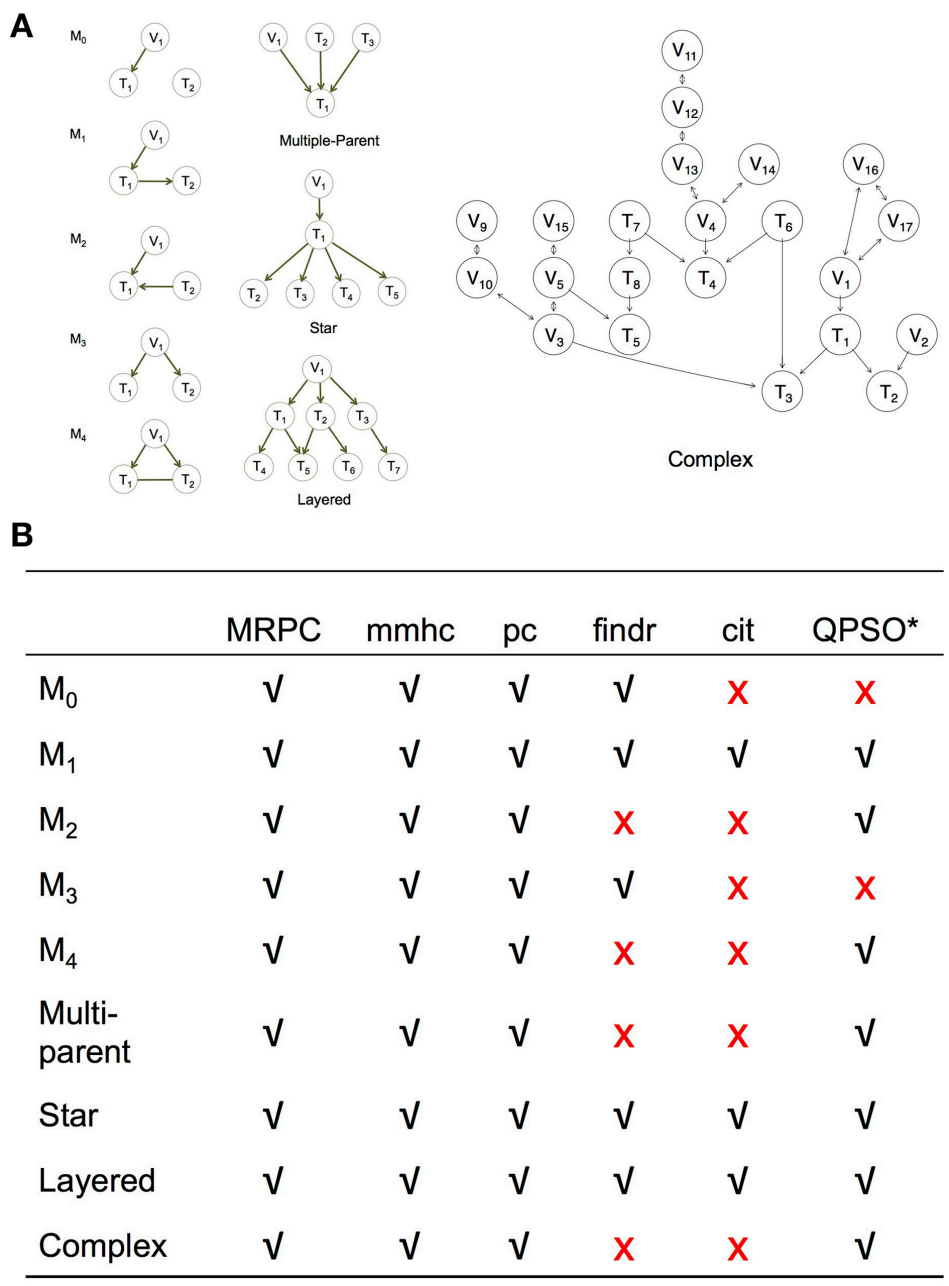
Simulation setup to compare MRPC with other methods. **(A)** Topologies used to generate synthetic data (section Generating Simulated Data). **(B)** Table summarizing graphs to which each method under comparison is applicable. *Note that QPSO does not learn the causal graph from scratch. Instead, it takes a graph skeleton as the input and seeks the optimal orientation of the edges in this undirected network. Edges involving genetic variants need to be already oriented in the skeleton. Therefore, QPSO does not identify M0 or M3.

We provide an example of the LOND method in Table S1, where values of δ_*i*_ and α_*i*_ for the first 18 tests of analysis of a simulated data set under the complex topology in Figure 3A are listed. The larger *a* is, the more conservative the LOND method, which means that fewer rejections will be made. We therefore used *a* = 2 throughout simulation and real data analyses.

### Robust Correlation in the Presence of Outliers

Genomic data may contain outliers (Badsha et al., 2013), which can greatly distort the inferred graph (see our simulation results below). Like the methods in pcalg, MRPC uses the correlation matrix as input. We implemented in MRPC a method for calculating the robust correlation matrix (Badsha et al., 2013) in place of Pearson correlation to alleviate the impact of outliers if they are present. Specifically, for data that are approximately normal (usually after preprocessing of the data), we calculated iteratively the robust mean vector **μ** and the robust covariance matrix ***v*** until convergence. At the *t*+1st iteration,

(1) $$\mu_{t+1}=\frac{\sum_{i=1}^{n}[\varphi_{\beta }\left({\text{x}}_{i};\;\mu_{t},{\text{v}}_{t}\right)\;{\text{x}}_{i}]}{\sum_{i=1}^{n}\varphi_{\beta }\left(\;{\text{x}}_{i};\;\mu_{t},{\text{v}}_{t}\right)}$$

and

(2) $$\begin{array}{r} v_{t+1}=\frac{\sum_{i=1}^{n}\left[\varphi_{\beta }\left(x_{i};\mu_{t},v_{t}\right)\left(x_{i}-\mu_{t}\right){\left(x_{i}-\mu_{t}\right)}^{T}\right]}{{\left(1+\beta \right)}^{-1}\sum_{i=1}^{n}\varphi_{\beta }\left(x_{i};\mu_{t},v_{t}\right)}, \end{array}$$

where,

(3) $$\begin{array}{r} \varphi_{\beta }\left(x;\mu ,v\right)=exp\left(-\frac{\beta }{2}{\left(x-\mu \right)}^{T}v^{-1}\left(x-\mu \right)\right). \end{array}$$

In the equations above, ***x***_*i*_ is the vector of gene expression in the *i*th sample, *n* the sample size, and β the tuning parameter. Equation (3) downweighs the outliers through β, which takes values in [0,1]. Larger β leads to smaller weights on the outliers. When β = 0, equation (2) is similar to the standard definition of the variance, except that the scalar is 1/*n*, whereas the unbiased estimator of the variance has a scalar of 1/(*n*-1). When the data matrix contains missing values, we perform imputation using the R package mice (van Buuren and Groothuis-Oudshoorn, 2011). Alternatively, one may impute the data using other appropriate methods, and calculate the correlation matrix as the input for MRPC.

When analyzing simulated data with no outliers, we set β = 0, which is close to Pearson correlation. We set β = 0.005 if outliers were included in simulation. On real data, we would usually perform two analyses with β = 0 and β = 0.005. These two values did not lead to different results in most cases.

### Generating Simulated Data

We simulated data using linear models for the five basic topologies, three common topologies in biology (Hunter, 2000; Alon, 2007) (such as multi-parent, star, and layered graphs), as well as a complex topology with over 20 nodes (Figure 3A). We varied the sample size, as well as the signal strength through the coefficients in the linear models.

In each topology, we simulated the data first for the nodes without parents, and then for other nodes. Genetic variants are nodes without parents, and we assume them to be biallelic with three genotypes 0, 1, and 2. Denote the minor allele frequency by *q* and assume Hardy-Weinberg equilibrium. Then the genotype of the *i*th variant, *V*_*i*_, follows a multinomial distribution:

$$\begin{array}{l} \mathrm{Pr}\left(V_{i}=0\right)={\left(1-q\right)}^{2},\mathrm{Pr}\left(V_{i}=1\right)=2q\left(1-q\right),\mathrm{Pr}\left(V_{i}=2\right)=q^{2}. \end{array}$$

Denote the *j*th molecular phenotype by *T*_*j*_ and the set of its parent nodes by P, which may be empty, or may include variant nodes or nodes of other molecular phenotypes. We assume that the molecular phenotype *T*_*j*_ follows a normal distribution

$$\begin{array}{l} T_{j}\sim N\left(\gamma_{0}+\sum_{k\in p}\gamma_{k}V_{k}+\sum_{l\in p}\gamma_{l}T_{l},\sigma_{j}^{2}\;\right). \end{array}$$

The variance may be different for different nodes. For simplicity, we use the same value for all the nodes.

We treat undirected edges as bidirected edges and interpret such an edge as an average of the two directions with equal weights. For example, for the undirected edge in Model 4 in Figure 1, we generate data for T_1_ → T_2_:

$$\begin{array}{l} T_{1}\sim N\left(\gamma_{0}\;+\gamma_{1}V,\sigma_{1}^{2}\right);\;T_{2}\sim N\left(\gamma_{0}\;+\gamma_{1}V+\gamma_{2}T_{1}{,\sigma }_{2}^{2}\;\right), \end{array}$$

and separately for T1←T2:

$$\begin{array}{l} T_{1}\sim N\left(\gamma_{0}\;+\gamma_{1}V+\gamma_{2}T_{2},\;\sigma_{1}^{2}\;\right);\;T_{2}\sim N\left(\gamma_{0}\;+\gamma_{1}V,\;\sigma_{2}^{2}\;\right). \end{array}$$

We then randomly choose a pair of values with 50:50 probability for each sample.

For simplicity in simulation, we set γ_0_ = 0 and all the other γ′s to take the same value, which reflects the strength of the association signal. We considered three values for the slopes: 0.2 (weak signal), 0.5 (moderate signal), and 1.0 (strong signal). We also varied the sample size: 50 (very small), 200 (small), 500 (medium), and 1,000 (large). Thus, we considered twelve combinations of signal strength and sample size (Tables S2, S3).

Under each combination, we generated 1,000 data sets for each topology. For each data set, which contains both genotype and gene expression data, we shuffled the columns corresponding to gene expression to generate one data set with those columns reordered; if an inference method is sensitive to the ordering of the columns, the inferred graph would have a large variance across data sets. We then applied each method to a data set with permuted columns.

The simulation strategy described above assumes the same signal strength (value of γ, the coefficient of the parent node) across the network, which allows us to examine the performance of the methods in simple and well-controlled settings. For the complex topology, we further allowed the values of γ to vary when generating data for each node. Each γ has equal probability of taking on one of three values: 0.2, 0.5 and 1.0. Similar to the procedure described above, we also generated 1,000 data sets with this strategy and applied relevant methods.

### Assessing the Inference Performance

To summarize the inference results, we computed the mean and standard deviation of recall and precision across 1,000 data sets for each method. Recall (i.e., power, or sensitivity) measures how many edges from the true graph a method can recover, whereas precision (i.e., 1-FDR) measures how many correct edges are recovered in the inferred graph:

Recall = (# edges correctly identified in inferred graph) / (# edges in true graph);

Precision = (# edges correctly identified in inferred graph) / (# edges in inferred graph).

An edge needs to be identified to be present before its direction determined. Therefore, we assign 1 to an edge with the correct direction and 0.5 to an edge with the wrong direction or no direction.

For all the topologies except M0 and M3 (which have no edge between T_1_ and T_2_), we excluded edges involving genetic variants when calculating recall and precision. This is because our method sets constraints on these edges such that as long as they are inferred to be present, they are always correctly oriented. Consider the scenario where the true graph is M1 (V → T_1_ → T_2_). Suppose that the inferred graphs are (i) V → T_1_ → T_2_, and V → T_2_; (ii) V → T_1_-T_2_; and (iii) V → T_1_←T_2_. We exclude edges V → T_1_ and V → T_2_ when calculating recall and precision. Then recall is 1/1 = 100%, 0.5/1 = 50%, and 0.5/1 = 50% for each of the three inferred graphs, respectively, whereas precision is identical to recall in this case.

For data simulated under the null hypothesis (M0 and M3), we assessed the inference performance through the empirical type I error rate, where we counted how many times an edge was inferred between T_1_ and T_2_, ignoring edges involving V.

Since each method under comparison had recall and precision over a large number of scenarios, we further summarized the performance of each method by the median of recall and precision across all topologies and all parameter settings. We also calculated for each method the standard deviation in recall and precision across all the scenarios.

### Application of Other Methods

We compared MRPC with two popular network inference algorithms, namely the Max-Min Hill Climbing (mmhc) method (implemented in bnlearn) and the pc method (implemented in pcalg), and three PMR-based methods, namely cit, findr, and QTL+Phenotype Supervised Orientation (QPSO; Wang and van Eeuwijk, 2014). Except for QPSO, which is implemented in MATLAB, all the methods are implemented in R.

We applied each of these methods to the (simulated or real) individual-level genotype and gene expression data matrix, where each row is an individual, and each column is a genotype or gene expression variable. mmhc uses the above data matrix as the input, whereas pc requires a correlation matrix derived from the above data matrix as the input. We further experimented with two settings of the pc function: the default (“PC”) and the conservative (“PCcons”). QPSO requires a graph skeleton as the input, which may be generated by pc or MRPC.

Unlike mmhc and pc that learn the graph skeleton first and orient the edges next, findr and cit test for directed edges in a single step for a triplet of nodes (the genetic variant and two gene expression nodes). This means that in order to learn the topology, we needed to examine all possible gene pairs (e.g., T_1_ and T_2_; and T_2_ and T_1_) and then apply findr or cit to the triplet of each of the gene pairs and the genetic variant. To keep track of the result for each gene pair, we used a K-by-K adjacency matrix, where K is the number of nodes. Parent nodes are in the rows and child nodes in the columns. The default values are 0, which corresponds to no edge between any node pair. Based on the hypothesis testing result from findr or cit, if there was evidence for a directed edge between two nodes, we added 1 to the current value in the adjacency matrix for those two nodes. Otherwise we left the value unchanged. After examining all gene pairs, we converted all positive values in the adjacency matrix to 1 to represent a directed edge. This way, no edges inferred would be eliminated in later tests. We then calculated recall and precision using the inferred adjacency matrix and that of the true graph, and averaged the rates across simulated data sets.

Although findr aims to compute a causality probability for a triplet, its current implementation for this calculation cannot be applied to small graphs, or cases where multiple genes share the same eQTL and where some of the genes do not have eQTLs. Specifically, we conducted five hypothesis tests (the p-values from these five tests are then converted to a causality probability) for each ordered gene pair with the genetic variant (using the function findr.pijs_gassist_pv() from the R package findr). Consider a triplet V_1_, T_1_ and T_2_. The null (H_0_) and alternative (H_a_) hypotheses of these five tests are:

Test #1: H_0_: V_1_ and T_1_ independent; H_a_: V_1_ → T_1_;
Test #2: H_0_: V_1_ and T_2_ independent; H_a_: V_1_ → T_2_;
Test #3: H_0_ (M1): V_1_ → T_1_ → T_2_; H_a_: V_1_ → T_1_, V_1_ → T_2_, T_1_ → T_2_;
Test #4: H_0_ (M0): V_1_ → T_1_, both independent of T_2_; H_a_: V_1_ → T_1_, V_1_ → T_2_, T_1_ → T_2_;
Test #5: H_0_ (M3): V_1_ → T_1_, V_1_ → T_2_; H_a_: V_1_ → T_1_, V_1_ → T_2_, T_1_ → T_2_.

We extracted the p values (i.e., *p*_*i*_, *i* = 1, …, 5) for the five tests. The data supports M0, if *p*_1_ is less than, and *p*_2_ and *p*_4_ greater than a certain threshold. The data supports M1, if *p*_1_ is less than, and *p*_3_ greater than a certain threshold. The data supports M3, if *p*_1_ and *p*_2_ are less than, and *p*_5_ greater than a certain threshold. We determined the *p*-value threshold with Bonferroni correction, dividing the desired threshold 0.05 by 5*m*, where *m* is the total number of genes pairs, and each findr test contains five tests.

cit generates an omnibus *p*-value for testing whether the triplet follows M1 (using the function cit.cp() from the R package cit). Similarly, we determine the *p*-value threshold also with Bonferroni correction (dividing 0.05 by the total number of genes pairs).

We also assessed the inference accuracy when the graph skeleton is known. In this case, the number of tests was reduced to one on simple models (M0, M1, and M3), to four in the star graph and to five in the layered graph. In other words, potential regulators and targets are known to findr and cit. For MRPC, however, we continued to assume that the skeleton was unknown.

### Accounting for Confounding Variables

Confounding variables may lead to biased inference of the topology. For example, if T_1_ and T_2_ in Model M3 (Figure 1) are both regulated by another gene, then the network might be wrongly inferred to be Model M4. Recently, Yang et al. (2017) developed the GMAC (Genomic Mediation analysis with Adaptive Confounding adjustment) method to explicitly account for confounding when inferring the causal relationship between the cis- and trans-target genes of the same eQTL. The confounding variable was included in the analysis as an additional node. Their analysis of real data indicated that Principal Components (PCs) from the entire gene expression matrix are reasonable candidates for confounding, as each PC is a linear combination of a large number of genes, and therefore represents a summary of potential impact from other genes outside the triplet.

In our application here, we also performed Principal Component Analysis (PCA) on the entire gene expression matrix, and used the top PCs as potential confounders. For a confounder to affect the causal inference, it should be associated with the genes of interest. We examined the association between each of the top 10 PCs and the eQTL-gene sets, identified statistically significant associations, and then applied MRPC jointly for the eQTL-gene set and the associated PC.

## Results

### MRPC Outperforms Existing Network Inference Algorithms and PMR-Based Methods on Synthetic Data in Overall Accuracy

For each topology, we generated 1,000 data sets with different combinations of signal strength and sample size, and ran MRPC, mmhc, pc, cit, findr, and QPSO with their default parameters wherever possible. We set the FDR level to be 0.05 for MRPC, the type I error rate for each test to be the default value of 0.05 for mmhc and pc, the FWER to be 0.05 for the Bonferroni correction when using cit and findr. Additionally, when applying MRPC, we used Pearson correlation (β = 0) and the LOND method with *a* = 2.

**Figure 4:**
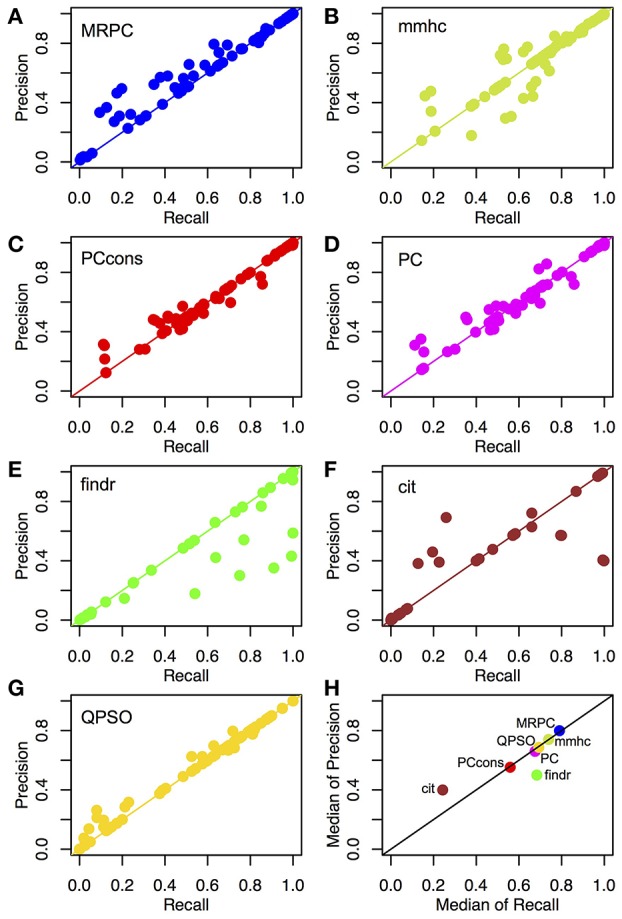
Recall and precision of different methods on simulated data. **(A–G)** Mean recall and precision averaged over 1,000 data sets simulated with four sample sizes and three signal strengths. see section Generating Simulated Data for simulation details. See Tables S2, **S3** for the mean and standard deviation of recall and precision from each method in each of the scenarios. **(H)** Median recall and precision over all parameter settings. We experimented with two settings of the pc function: the default (“PC”) and the conservative (“PCcons”). Since the default setting outperforms the conservative one, we generally use only the default setting in other analyses. Note that only 20 datasets were used for QPSO in each parameter setting due to long runtime.

We compared the recall and precision across these methods. For any method, the two rates generally improved with a stronger signal strength or a larger sample size (Tables S2, S3). As explained in section Assessing the Inference Performance, recall measures power and precision is 1-FDR. High recall does not suggest high precision, and vice versa. In our simulation study here, however, most scenarios examined small graphs, and the numbers of true and inferred edges were low. As a result, the denominators in recall and precision tended to be close, and the rates of recall and precision tended to be similar (Figures 4A–G). We therefore further summarized the two metrics with the median rates across all scenarios under comparison (Figure 4H).

Across different topologies and parameter settings, MRPC had the highest median recall and precision, with both median recall and median precision above 82%. MRPC was followed by mmhc, QPSO, pc with two parameter settings, findr, with cit trailing far behind (Figure 4H; Tables S2, S3). MRPC recovered the true graph particularly well at moderate or stronger signal with a medium or larger sample size. For the complex topology, MRPC performed consistently better than pc and mmhc. This is still the case when the signal strength was heterogeneous across the complex topology (Figure S3). To understand what caused low recall or low precision, we examined some of the inferred graphs from different methods and observed that pc could be unable to determine edge directions or could wrongly identify v-structures when the true model contained none (Figures S4–S6). Meanwhile, PMR-based methods, such as findr and cit, could infer too many or too few edges, whereas QPSO could not identify the direction correctly (Figures S4–S6). We also summarized the median standard deviation of recall and precision in Figure S7. Note that the standard deviation in recall and precision reflected variation due to both different data sets and different node orderings. Except for QPSO, the methods under comparison did not differ much in variation.

We next investigated the performance of these methods when the true graph is M0 or M3, neither of which contains an edge between the two gene nodes T_1_ and T_2_. These two graphs are two types of nulls: T_1_ and T_2_ have no marginal or conditional dependence in M0, whereas they have marginal but not conditional dependence in M3. We simulated 1,000 data sets as described in section Generating Simulated Data for different settings and applied each of the methods (except for cit and QPSO; Figure 3B). Since it is not sensible to calculate recall or precision for these nulls, we calculated the rate of the edge T_1_-T_2_ being inferred, which serves as the empirical type I error rate (Table S4). As expected, this rate generally decreased as the signal strengthened. When the sample size increases, however, any statistical test is more likely to reject the null, thus the empirical type I error rate does not necessarily have a linear relationship with the sample size. Overall, findr with the Bonferroni correction was the most conservative, with MRPC being close. The mmhc and pc method had higher error rates, although the rates were generally lower than 0.05, which was the default type I error rate set for each test in these methods. This is rather interesting, as both mmhc and pc involved multiple tests for M0 and M3. This illustrates why controlling the error rate is challenging in network inference, when the total number of tests is unknown beforehand, and when multiple tests may be performed on the same edge.

We further assessed the performance in the presence of outliers. Here, we focused on the complex topology and replaced 10 data points in each simulated data set with data generated from a uniform distribution. We applied MRPC with robust correlation (β = 0.005), as well as mmhc and pc with the default parameters as before. We also calculated recall and precision for the inferred graphs. MRPC with robust correlation substantially outperformed pc and mmhc (Figure S8).

In terms of speed, our method can be 2–40 times faster than cit and 2–3 orders of magnitude faster than QPSO, but 3–8 times slower than mmhc, 7–20 times slower than pc, and 1–2 orders of magnitude slower than findr (see runtime comparison in Table S5). Since QPSO is at least an order of magnitude slower than other methods, we calculated recall and precision only for 20 (instead of 1,000) data sets in simulation for QPSO, which therefore had a larger standard deviation in recall and precision as explained above (Figure S7).

### Existing PMR-Based Methods Cannot Deal With Complex Causal Relationships

We examine the performance of PMR-based methods more closely in this section. Since cit and findr focus on Model 1, the topologies they can identify are limited to those that involve primarily Model 1, such as the star graph and the layered graph: the star graph consists of four M1s, and the layered graph five (Figure 3A). The findr method can also be used to identify M3.

Unlike MRPC, which is agnostic about which genes may be potential regulators and which potential targets, findr and cit are applied to ordered gene pairs iteratively, requiring specification of which of the two genes is the potential regulator and which the target. For example, to test whether the data are simulated under M1, then findr and cit will be performed twice, on (V_1_, T_1_, T_2_) and then on (V_1_, T_2_, T_1_). The number of ordered gene pairs is $2\times \left(\overset{5}{2}\right)=20$ for the star graph and $2\times \left(\overset{7}{2}\right)=42$ for the layered graph. We applied Bonferroni correction with a family wise type I error rate of 0.05. Take again the star model with a sample size of 1,000 for example. Although Bonferroni correction is already a conservative method for multiple testing, findr still sometimes inferred more edges than there are (summarized by the lower precision in Figure 4H), whereas cit could infer a very dense graph or no edges at all (summarized by low recall and low precision in Figure 4H; also see examples in Figures S4–S6). Even when the graph skeleton was known, findr and cit still did not outperform MRPC in nearly all cases (Figures S9, S10).

Similar to MRPC, QPSO also has connections to PC algorithms. However, QPSO does not infer a graph skeleton. Instead, it requires a graph skeleton as the input and seeks the optimal orientation of the edges, its performance therefore depending heavily on how well the skeleton is inferred. Whereas, the authors of QPSO used pc to generate the skeleton, we used MRPC to generate the input, having observed the unsatisfactory performance of pc. With a more accurate skeleton, QPSO is still lacking both in recall and in precision in general (Figure 4H).

### Application of MRPC to Distinguishing Direct and Indirect Targets of eQTLs Analysis of the GEUVADIS Data

A single eQTL can be statistically associated with the expression of multiple genes. For such eQTLs, we are interested in identifying true targets and understand how multiple targets regulate one another. Multiple genes are potential targets often because these genes are physically close to one another on the genome, and the eQTL analysis usually examines the association between one Single Nucleotide Polymorphysim (SNP) and one gene at a time, ignoring dependence among genes.

The GEUVADIS project (Lappalainen et al., 2013; see **Web Resources**) performed RNA-seq (gene expression) in Lymphoblastoid Cell Lines (LCLs) on a subset of individuals from the 1,000 Genomes Project, including 373 Europeans. Combining the gene expression data with the genotype data from the 1,000 Genomes Project, the GEUVADIS project identified eQTLs across the human genome. Among the most stringent set of eQTLs, 62 have more than one target gene.

**Figure 5:**
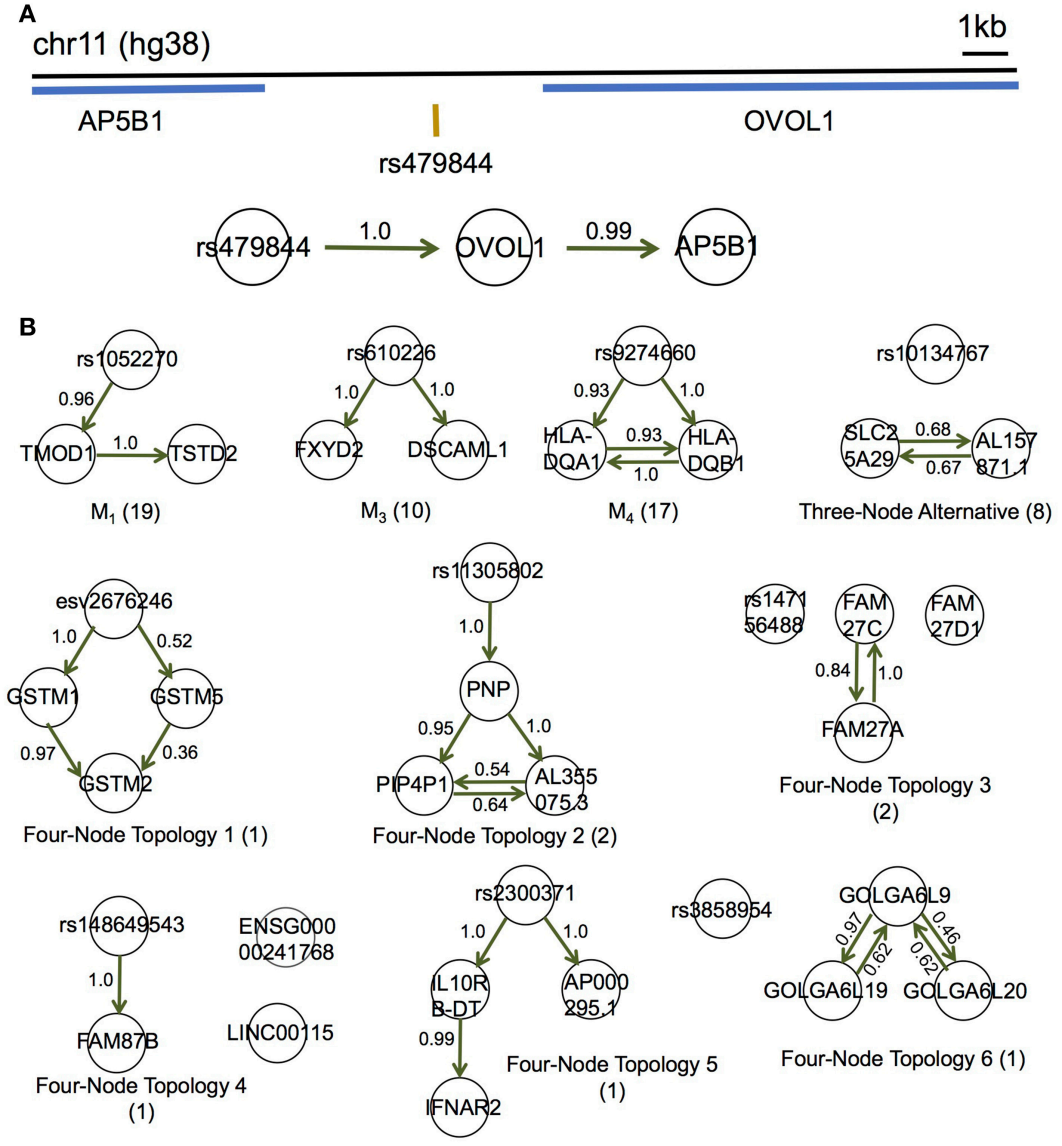
MRPC distinguishes direct and indirect target genes of eQTLs in the GEUVADIS data for the European cohort. **(A)** rs479844 is a GWAS significant SNP for atopic march in the GWAS Catalog, and an eQTL identified in GEUVADIS for two genes. **(B)** MRPC learns 10 distinct topologies among associated genes for eQTLs. Numbers on edges are proportions of the corresponding directed edge being present in a bootstrap sample of 200. The number in parentheses under each topology is the number of eQTL-gene sets with the corresponding inferred topology.

We applied MRPC to each of these eQTLs and their associated genes in the 373 Europeans, and identified 10 types of topologies (Figure 5; Table S6; also see comparison with mmhc and pc for some of the eQTL-gene sets in Figure S11). Three of these 10 types were Models 1, 3, and 4 shown in Figure 1. Seven other topologies were identified for eight eQTLs each with three associated genes (Table S6).

Although the multiple associated genes of the same eQTL are physically near one another, our method showed promise in teasing apart the different dependence (or regulatory relationships) among these genes. For example, the SNP rs479844 (chr11:65,784,486; GRCh38), one of the 62 eQTLs, is significant in at least three GWASs for atopic march and more specifically, atopic dermatitis (*p*- values ranging from 10^−10^ to 10^−18^) (Paternoster et al., 2011, 2015; Marenholz et al., 2015; MacArthur et al., 2017). This SNP has been linked with two genes, *AP5B1* (chr11:65,775,893-65,780,802) and *OVOL1* (chr11:65,787,022-65,797,219), in these GWASs, but it is unclear which is the real target. Our MRPC inferred Model 1 for the triplet: rs479844 → *OVOL1*→*AP5B1* (Figure 5A), which suggests that *OVOL1* is more likely to be the direct target, and *AP5B1* the indirect one. Meanwhile, for *HLA-DQA1* (chr6:32,637,403-32,654,846) and *HLA-DQB1* (chr6:32,659,464-32,666,689), both genes are associated with the SNP rs9274660 and located in the major histocompatibility (MHC) region of high linkage disequilibrium. As expected, MRPC inferred an undirected edge between the two genes, as the information on the two genes is highly symmetric in the genotype and gene expression data. By contrast, mmhc and pc often mis-specified edges or their directions (Figure S11).

We performed bootstrap to assess the accuracy in inferred edges (Figure 5B). For each eQTL-gene set, we resampled the individuals with replacement 200 times, and applied MRPC to each of the bootstrap samples. We averaged all the topologies (represented by adjacency matrices) such that the resulting matrix contained proportions of the corresponding edges being present. These proportions are estimated probabilities of the edge being present. Most of these bootstrap probabilities are above 50%, providing support to the inferred networks.

### Examining the Impact of Confounding

We performed PCA on the expression of all the genes among European samples in the GEUVADIS consortium, and examined whether top PCs are correlated with the eQTLs. If not, then Assumption (1) in the PMR is satisfied. Note that in this paragraph the abbreviation PC refers to principal component, not the algorithm. We calculated Pearson correlation between each of the top 10 PCs and each of the eQTLs and genes (194 variables in total) on which we applied MRPC (Figure S12), and tested whether these pairwise correlations were statistically significant (FDR = 0.05 with the q value method). Among the 186 significant correlations, none was between a PC and an eQTL. To further examine the impact of the PCs on genes, we selected from the 186 significant correlations the top 98 based on the magnitude of the correlation (|r|>0.3), which involved 32 eQTL-gene sets and 7 PCs. Since a PC could be associated with one or more genes in an eQTL-gene set, we applied MRPC to each of these 32 eQTL-gene sets together with the associated PCs, and examined whether incorporating the PCs changed the inference of the topologies. PCs may be associated with the genes in different ways, but none of them changed the topology inferred for the eQTL-gene sets (Figure S13).

### Replication Analysis Using GTEx

Since the GTEx consortium (The GTEx Consortium, 2017) contains data also from LCLs, we next examined whether the causal relationships inferred from the GEUVADIS data may be replicated in the LCL samples from GTEx.

The GTEx consortium has profiled genotypes and gene expression levels in 53 tissues across 714 donors (Release V7, dbGaP Accession phs000424.v7.p2; https://www.gtexportal.org/home/). We extracted the gene expression data of the LCLs, and the genotype data of the eQTLs used in the GEUVADIS analysis. We applied PEER (probabilistic estimation of expression residuals) normalization (Stegle et al., 2012) (with 15 PEER factors) to the gene expression (following the procedure provided by the authors of the PEER package at https://github.com/PMBio/peer/wiki/Tutorial), such that the expression data were processed in the same way as the GEUVADIS gene expression data. Since GTEx uses chromosome locations to identify genetic variants, we extracted the coordinates of the GEUVADIS eQTLs in Ensemble (GRCh 37; https://grch37.ensembl.org/index.html) using the rs IDs of the eQTLs. Not all GEUVADIS eQTLs can be found in the GTEx samples. Among eQTLs that can be found in the GTEx samples, not all their associated genes have expression measurements. In the end, we found 46 eQTL-gene sets with data available in both GEUVADIS and GTEx LCLs (Table S6). For each of these sets, we ran MRPC with an FDR of 0.05, and summarized the results in Tables S7–S11. For those sets that were inferred to have an M1 model by MRPC in GEUVADIS, we also ran findr and cit on each set to test whether there is a causal model as in V_1_ → T_1_ → T_2_ or V_1_ → T_2_ → T_1_ (Table S7).

However, the sample size of 117 is much smaller in the GTEx LCL samples, which reduces the expected number of causal relationships to be replicated. We therefore took two approaches in the replication analysis: the first approach was applying MRPC directly to the GTEx sample of each eQTL-gene set. If the topology inferred from the GEUVADIS data was not replicated, we applied the second approach of resampling from the GTEx sample with replacement, such that the sample size became comparable to GEUVADIS. We then applied MRPC to the resampled GTEx data. The latter approach is justifiable here because we often observed similar correlation patterns between GEUVADIS and GTEx for the same eQTL-gene set, suggesting that the two data sets carry similar information about the gene network. Simply because the sample size in GTEx is lower than that in GEUVADIS, MRPC tended to infer fewer edges from GTEx than from GEUVADIS, and the resulting topology was often part of the topology inferred from GEUVADIS.

For eQTL-gene sets that were inferred to have an M1 model in GEUVADIS by MRPC, we applied findr and cit, in addition to MRPC, for comparison. Fourteen eQTL-gene sets with an M1 model have the genotype and gene expression data in both GEUVADIS and GTEx LCL samples. MRPC replicated the topology for 12 sets, findr 13 and cit only 5 (without and with up-sampling; Table S7), consistent with our expectation based on our simulations (Figure 4H): whether the graph skeleton is known or not, MRPC and findr had similar performance on M1 across different sample sizes and signal strengths, both methods being much better than cit. In particular, we replicated the relationship rs479844 → *OVOL1*→*AP5B1* with both MRPC and findr in the GTEx LCL samples.

Among eQTL-gene sets with other MRPC-inferred topologies, nine sets inferred to have an M3 model also had data in GTEx and seven of them were replicated by MRPC (Table S8). Sixteen sets inferred to have an M4 model also had data in GTEx and six were replicated (Table S9). Four sets inferred to have three-node alternative topology (Figure 5B) had data in GTEx and all of them were replicated (Table S10). Among the eight sets involving three genes, three sets had data in both GEUVADIS and GTEx, and one was replicated (Table S11). However, most of the edges in these sets had high bootstrap probabilities, and MRPC inference for these sets was not affected by potentially confounding variables.

**Figure 6:**
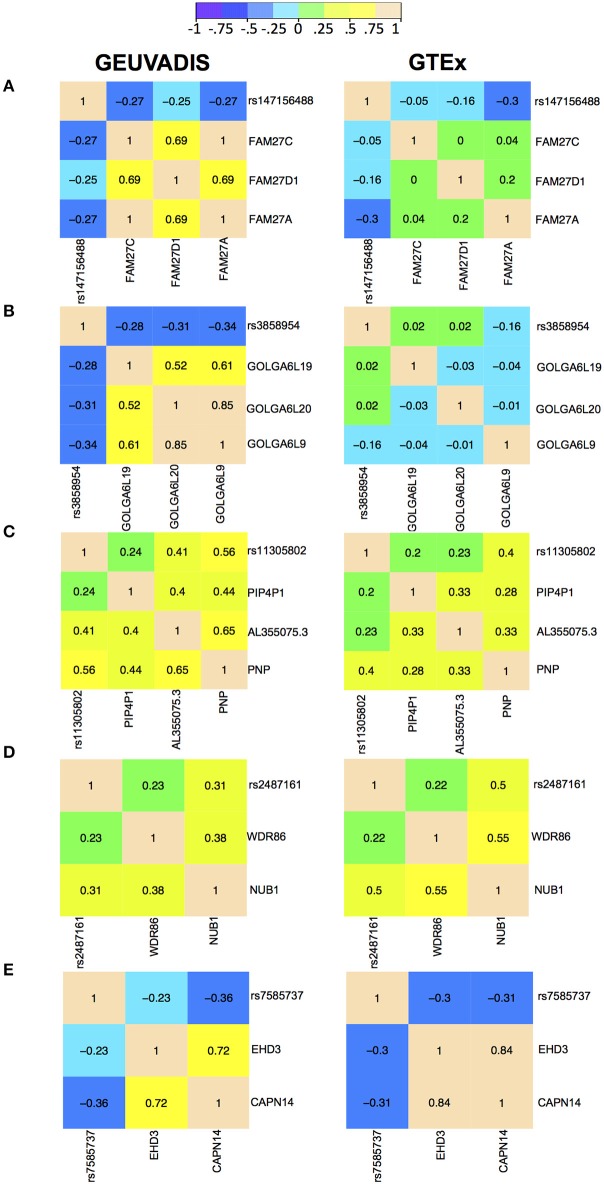
Correlation heatmaps of five eQTL-gene sets using the GEUVADIS data and independently using the GTEx data. Both data sets have been PEER normalized. The top two sets were not replicated in GTEx: **(A)** SNP rs147156488, and **(B)** SNP rs3858954. The bottom three sets were replicated in GTEx: **(C)** SNP rs11305802 (replicated with upsampling), **(D)** SNP rs2487161 (replicated without upsampling), and **(E)** SNP rs7585737 (replicated with upsampling).

**Figure 7:**
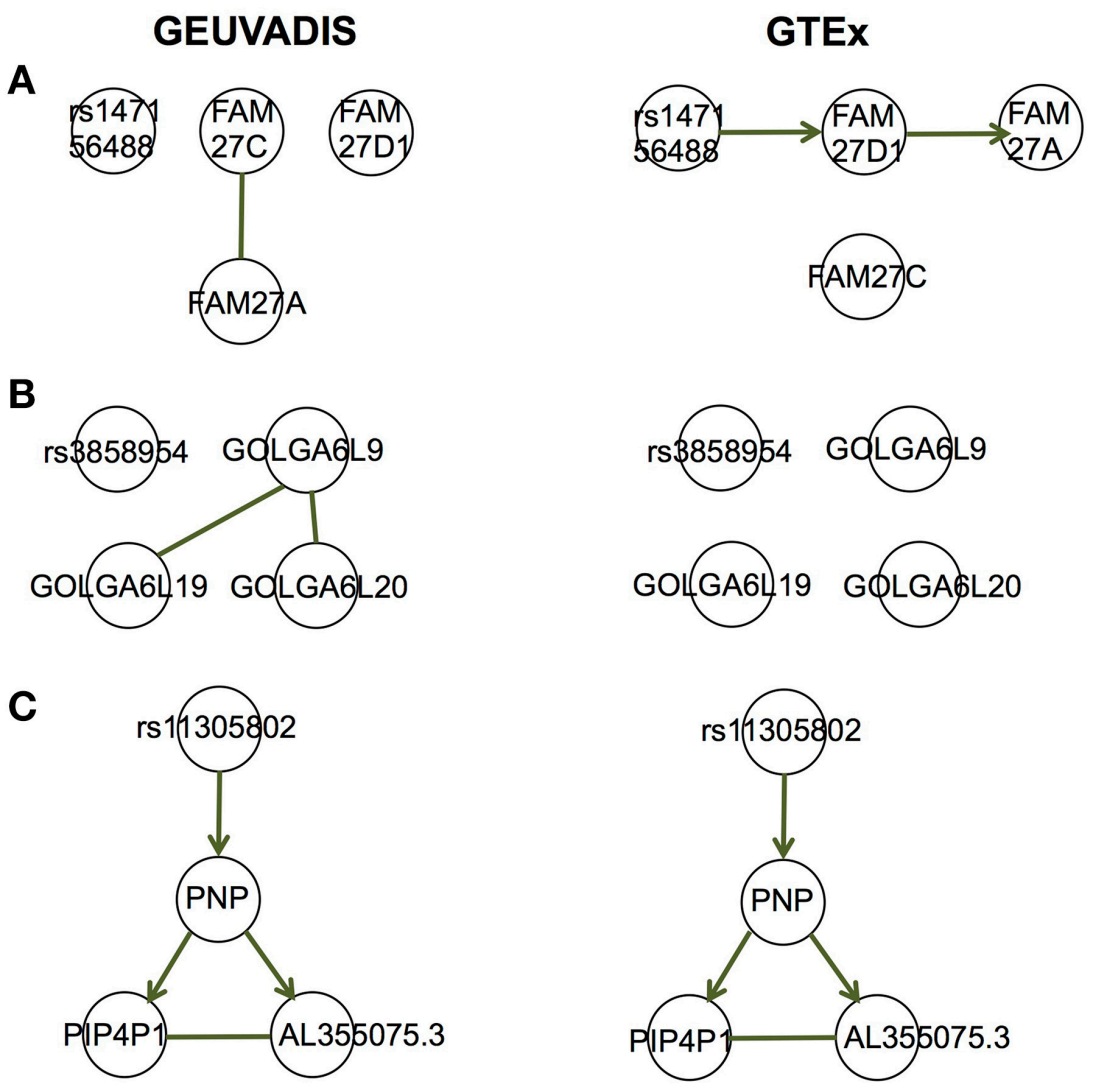
Topologies inferred by MRPC on GEUVADIS and GTEx data for three eQTL-gene sets in Figures 6A–C. Three target genes have been identified for each of these eQTLs. When the correlation patterns were qualitatively different in the two consortia for the first two sets **(A, B)**, MRPC could not replicate the topologies but instead produced graphs consistent with the correlation patterns. On the other hand, when the correlation patters were similar **(C)**, MRPC replicated the topology. Upsampling was used in the MRPC inference for the GTEx data to compensate for a smaller sampler size.

For the eQTL-gene sets whose topologies were not replicated in GTEx, it turned out that the correlation patterns were vastly different in these sets between the two consortia, even when the ethnicity (European), tissue type (LCLs) and data normalization method (PEER normalization on the gene expression data) were the same (see examples in Figure 6). For these genes, the overall correlation patterns are much weaker in the GTEx data than in the GEUVADIS data (Figures 6A,B). In contrast, the correlation patterns in eQTL-gene sets whose topologies were replicated in GTEx are similar in the two consortia (Figures 6C–E). One possible explanation for the differences in correlation patterns of some genes is measurement errors, in particular, the type of measurement error that may be gene-specific. Additional investigations are needed to understand these errors, and better normalization or estimation methods are needed to systematically adjust for these errors, such that more accurate estimates of correlations are available not only for causal inference, but also for other analyses based on correlations. On the other hand, these correlation heatmaps further confirmed that our MRPC inference is consistent with the correlation patterns: if the correlation patters are similar in the two data sets, then MRPC will infer the same topology (Figure 7).

## Discussion

In summary, we have developed MRPC to infer causal networks. Our MRPC method examines a variety of causal relationships implied by the PMR, and takes advantage of the development of machine learning algorithms for causal graph inference. MPRC integrates genotypes with molecular phenotypes, and can efficiently and accurately learn causal networks. Our method is flexible as it requires only the genotype data (SNPs or other types of variants) and the molecular phenotype measurements (gene expression, or other functional data, such as exon expression, RNA editing, DNA methylation, etc.), and can be applied to a wide range of causal inference problems. Our method is also non-parametric in that no explicit distributions are assumed for the underlying graph. MRPC uses individual-level genomic data to learn plausible biological mechanisms from combining genotype and molecular phenotypes.

Two features distinguish MRPC from existing methods. First, MRPC accounts for all possible causal relationships that a triplet with a genetic variant can have under the PMR. This extended interpretation of the PMR allows MRPC to go beyond the canonical causal model examined by other PMR-based methods and can deal with networks of realistic causal relationships. Computationally, incorporation of the PMR puts constraints on the space of possible graphs and allows for efficient search of graphs consistent with the data. Second, MRPC implements an online FDR control method (the LOND method), which controls the overall error rate and helps reduce false positives. To our knowledge, this is the first time an online FDR control method is incorporated into a network inference method. General methods implemented in bnlearn and pcalg for learning DAGs do not control the overall error rate; instead, they control the type I error rate only for each individual test no matter how many tests are conducted. On the other hand, in cit and findr, one may control the FWER with Bonferroni correction or the overall FDR with regular FDR control methods (e.g., the Benjamini-Hochberg method, the q value method, etc.). However, this is because cit and findr can infer only one or two causal relationships. The total number of hypothesis tests can therefore be known beforehand. As our simulation demonstrated, false positive edges are a severe problem in other methods, whether they are based on the PMR or not. MRPC with the LOND method showed promising performance in reducing false positives, although it still needs much improvement in order to have better control of the overall error rate, especially when the signal is weak, or when the sample size is small. Indeed, the comparison of recall, precision and type I error in our simulation study showed that it is a challenge to control the overall error rate (FDR or FWER) in network inference, not only because the control has to be done in an online manner (with the total number of tests being unknown beforehand), but also because of the dependence among tests, as well as different types of dependence induced by different topologies (Ramdas et al., 2019).

Although our MRPC employs the PMR, it is fundamentally different from other PMR-based methods. Most of the methods incorporating the PMR fall into two classes. One class, including cit and findr, is called *mediation-based methods* that require individual-level data, generally do not estimate the causal effect sizes, and can infer networks of multiple phenotypes (e.g., a network of gene expression). The other class of methods are called *MR methods* (Hemani et al., 2017) that can be applied to individual-level data as well as summary statistics, estimate the causal effect sizes, and generally focus on three-node graphs with one node being the genetic variant, and the other two nodes being phenotypes of interest. Both classes of methods employ the PMR and focus on the canonical causal model, in which exposure acts as the mediator. Although our MRPC method is closer to the mediation-based methods according to the characteristics described above, the notion of “mediation” is less relevant to our method; only Model 1 considers the canonical causal model, and therefore one of the two genes acts as the mediator (Figure 1). More importantly, with our method we consider the PMR as a way to define plausible causal relationships and to put constraints on the space of possible graphs. As a result, our method can recover a variety of causal relationships, instead of the few that other PMR-based methods can identify (Figure 3B).

Built on the PC algorithm, MRPC also enjoys the statistical properties of this algorithm in inferring causal graphs. A causal graph with a mixture of directed and undirected edges is essentially an equivalent class of directed acyclic graphs (DAGs) that have the same likelihood. However, the search problem of learning the DAG with the highest likelihood when the number of parent nodes is >1 has been proven to be NP-complete (Hoffgen, 1993), the hardest computational problem; NP stands for non-deterministic polynomial time. An NP-complete problem means that i) its solution may be verified in polynomial time although the solution is difficult to determine, and ii) similar problems may be reduced to this problem in polynomial time. Learning even just the equivalent classes of a DAG with the number of parent nodes being >1 is also NP-complete (Chickering, 1996), as the space of equivalent classes of DAGs is super-exponential (Kalisch and Bühlmann, 2007) in the number of nodes. Therefore, the PC algorithm and similar algorithms get around the computational issue with local searches. Although it is not known theoretically that these PC algorithms achieve the global optimality defined by, for example, the likelihood, it has been shown that the PC algorithm is consistent (Kalisch and Bühlmann, 2007): with a large sample size, the PC algorithm is expected to recover the true graph. In particular, consistency of the PC algorithm is essentially consistency of the step of graph skeleton inference, as this step contains all the statistical inference (Meek, 1995). Since MRPC uses essentially the same procedure for skeleton inference as the PC algorithm, MRPC is also consistent.

As general-purpose DAG inference methods, mmhc and pc allow for directions of certain edges to be fixed before inference. However, this option also means that the corresponding edges are guaranteed to be in the inferred graph, even if the data provides no evidence for such presence. This is particularly problematic in our analysis of the GEUVADIS data, as in each eQTL-gene set, all the genes are targets with strong association with the eQTL; otherwise, these genes would not have been identified to be targets. Using mmhc and pc with fixed edge directions will result in graphs with edges connecting the eQTL with all the genes, when our goal is to distinguish which of these edges may be explained away by other genes. This further illustrates the flexibility of our MRPC method, which fixes directions of certain edges while still assessing the evidence in the data for the presence of these edges.

MRPC currently does not directly deal with multiple genetic variants associated with the same molecular phenotype. For network inference, we recommend using the variant with the strongest association, or merging the multiple variants to create a haplotype variant with the haplotypes being the new genotypes (e.g., two SNPs in linkage disequilibrium, each having three genotypes, can be merged into one variant with genotypes 00, 01, 02, 10, 11, 12, 20, 21, and 22).

Here, we demonstrated the outstanding performance of MRPC on small to moderately-sized graphs. Additional work is needed to extend the ability of MRPC to larger graphs while retaining inference accuracy. Indeed, apart from mmhc and pc, other existing methods for inferring large causal graphs also tend to have high false positive rates: for example, the TRANSWESD (TRANSitive Reduction in WEighted Signed Digraphs; Flassig et al., 2013) method developed for the DREAM5 (Fifth Dialogue on Reverse Engineering Assessment and Methods) Systems Genetics Challenge A (a network of 1,000 SNPs and 1,000 genes with directed and undirected edges) showed better performance than other participating method for this challenge. However, even TRANSWESD has an actual FDR as high as 64% at a large sample size of 999, suggesting that much work is still needed to accurately infer a large causal graph.

Like most PMR-based methods, we assume that the three assumptions of the PMR are satisfied (see Introduction). Whereas, Assumptions (i) and (ii) are easier to establish through stringent tests for association between genetic variants and genes, Assumption (iii) is harder to achieve. Here, we took an approach inspired by the GMAC method (Yang et al., 2017) to examine the impact of confounding variables on the inferred graphs. As the next step, we are working on extensions of MRPC to more systematically and explicitly account for confounding variables during the causal network inference.

## Web Resources

MRPC: https://cran.r-project.org/web/packages/MRPC/index. html

pcalg: https://cran.r-project.org/web/packages/pcalg/index.html

bnlearn: https://cran.r-project.org/web/packages/bnlearn/index.html

cit: https://cran.r-project.org/web/packages/cit/index.html

findr: https://github.com/lingfeiwang/findr

GTEx: https://www.gtexportal.org/home/datasets

GEUVADIS: http://www.ebi.ac.uk/Tools/geuvadis-das/

## Data Availability

Publicly available datasets were analyzed in this study. This data can be found here: GTEx: https://www.gtexportal.org/home/datasets; GEUVADIS: http://www.ebi.ac.uk/Tools/geuvadis-das/

## Author Contributions

AF designed the project. MB and AF developed the method. MB implemented the software. MB and AF performed all the analyses and wrote the manuscript.

### Conflict of Interest Statement

The authors declare that the research was conducted in the absence of any commercial or financial relationships that could be construed as a potential conflict of interest.
