# Supplementary material for: Learning Causal Biological Networks With the Principle of Mendelian Randomization
Source: Front Genet. 2019 May 21;10:460. doi: 10.3389/fgene.2019.00460 (PMC6536645; doi:10.3389/fgene.2019.00460)
Supplement: Supplementary file 1 [file Data_Sheet_1.pdf]

Input:
- Correlation matrix of the data matrix, which is N-by-(Q+M) for N individuals, genotypes at Q variants and expression of M genes. May use robust correlation.
- FDR threshold.

Output:
- A graph with directed and undirected edges.

Algorithm:
1. Learning the graph skeleton (i.e., all edges are undirected).
   1) Initialization:
      A fully connected graph for all the nodes (i.e., variants and molecular phenotypes).
   2) Statistical tests of marginal independence for all pairs of nodes. Remove the edge when the p value is above the sequential-test FDR threshold.
   3) For node pairs still connected with an edge, perform conditional independence tests, conditioning on one other node, two other nodes, and so on. Remove the edge when the p value is above the sequential-test FDR threshold.
2. Inferring the direction of the edges in the graph skeleton.
   1) Edges involving the variants always go from the variants to the molecular phenotype. If an edge connects two variants, then the edge remains undirected (or equivalently bidirected).
   2) Identifying the v-structure (e.g., $T_1 \rightarrow T_2 \leftarrow T_3$):
      - Identify triplets T_1—T_2—T_3.
      - Check whether a test has been performed for independence between T_1 and T_3 given T_2. If so and if the p value is significant, then the directions of the two edges are both pointing to T_2. If such test has not been performed, run this test, while controlling the overall FDR with the LOND algorithm. If the p value is not significant, then no direction is assigned to the two edges.
   3) Orienting remaining edges by forming triplets and applying the PMR:
      - Identify triplets with at least one directed edge. The node with the outgoing edge will play the role of a genetic variant.
      - Check which one of the basic topologies in **Fig. 1** (main text) is consistent with the triplet, using results from previous conditional independence tests.
      - If none of the basic topologies describe the triplet, then leave the edge of interest as undirected (or equivalently, bidirected).
      - Iterate through all edges until no edges can be oriented.

Figure S1: The detailed MRPC algorithm. See **Figure S2** for an example of Step II.

Undirected (symmetric) adjacency matrix after Step I (see graph skeleton in **Figure 2A (2)** in the main text):

	V_1	V_2	T_1	T_2	T_3	T_4
V_1	0	1	1	0	0	0
V_2	1	0	0	0	0	0
T_1	1	0	0	1	1	0
T_2	0	0	1	0	0	1
T_3	0	0	1	0	0	0
T_4	0	0	0	1	0	0

Step II:
Initialize directed (asymmetric) adjacency matrix:

					Child Node		
		V_1	V_2	T_1	T_2	T_3	T_4
	V_1	0	0	0	0	0	0
	V_2	0	0	0	0	0	0
Parent	T_1	0	0	0	0	0	0
Node	T_2	0	0	0	0	0	0
	T_3	0	0	0	0	0	0
	T_4	0	0	0	0	0	0

Current list of edges to be oriented: V_1-V_2, V_1-T_1, T_1-T_2, T_1-T_3, T_2-T_4.

1) **Edges involving genetic variants**: V_1-V_2, V_1-T_1.
   Oriented as: $V_1 \leftrightarrow V_2$, $V_1 \rightarrow T_1$.
   Current directed adjacency matrix:

					Child Node		
		V_1	V_2	T_1	T_2	T_3	T_4
	V_1	0	1	1	0	0	0
	V_2	1	0	0	0	0	0
Parent	T_1	0	0	0	0	0	0
Node	T_2	0	0	0	0	0	0
	T_3	0	0	0	0	0	0
	T_4	0	0	0	0	0	0

   Current list of edges to be oriented: T_1-T_2, T_1-T_3, T_2-T_4.

2) **Potential v-structures**:
   $V_1 \rightarrow T_1 – T_2$: conditional independence test rejected, therefore v-structure $V_1 \rightarrow T_1 \leftarrow T_2$;
   $V_1 \rightarrow T_1 – T_3$: conditional independence test not rejected, therefore not v-structure;
   $T_1 – T_2 – T_4$: conditional independence test not rejected, therefore not v-structure.
   Current directed adjacency matrix:

		Child Node					
		V_1	V_2	T_1	T_2	T_3	T_4
	V_1	0	1	1	0	0	0
	V_2	1	0	0	0	0	0
Parent Node	T_1	0	0	0	0	0	0
	T_2	0	0	1	0	0	0
	T_3	0	0	0	0	0	0
	T_4	0	0	0	0	0	0

Current list of edges to be oriented: T_1-T_3, T_2-T_4.

3) **Remaining edges in triplets**:
$V_1 \rightarrow T_1 – T_3$: matching Model 1: $V_1 \rightarrow T_1 \rightarrow T_3$;
$T_1 \leftarrow T_2 – T_4$: not matching any of the five basic models; leave $T_2 – T_4$ as undirected (equivalently bidirected).
Current directed adjacency matrix:

		Child Node					
		V_1	V_2	T_1	T_2	T_3	T_4
	V_1	0	1	1	0	0	0
	V_2	1	0	0	0	0	0
Parent Node	T_1	0	0	0	0	1	0
	T_2	0	0	1	0	0	1
	T_3	0	0	0	0	0	0
	T_4	0	0	0	1	0	0

Current list of edges to be oriented: empty.

Final adjacency matrix of the causal graph:

		Child Node					
		V_1	V_2	T_1	T_2	T_3	T_4
	V_1	0	1	1	0	0	0
	V_2	1	0	0	0	0	0
Parent Node	T_1	0	0	0	0	1	0
	T_2	0	0	1	0	0	1
	T_3	0	0	0	0	0	0
	T_4	0	0	0	1	0	0

Figure S2: An example of details of Step II of the MRPC algorithm.

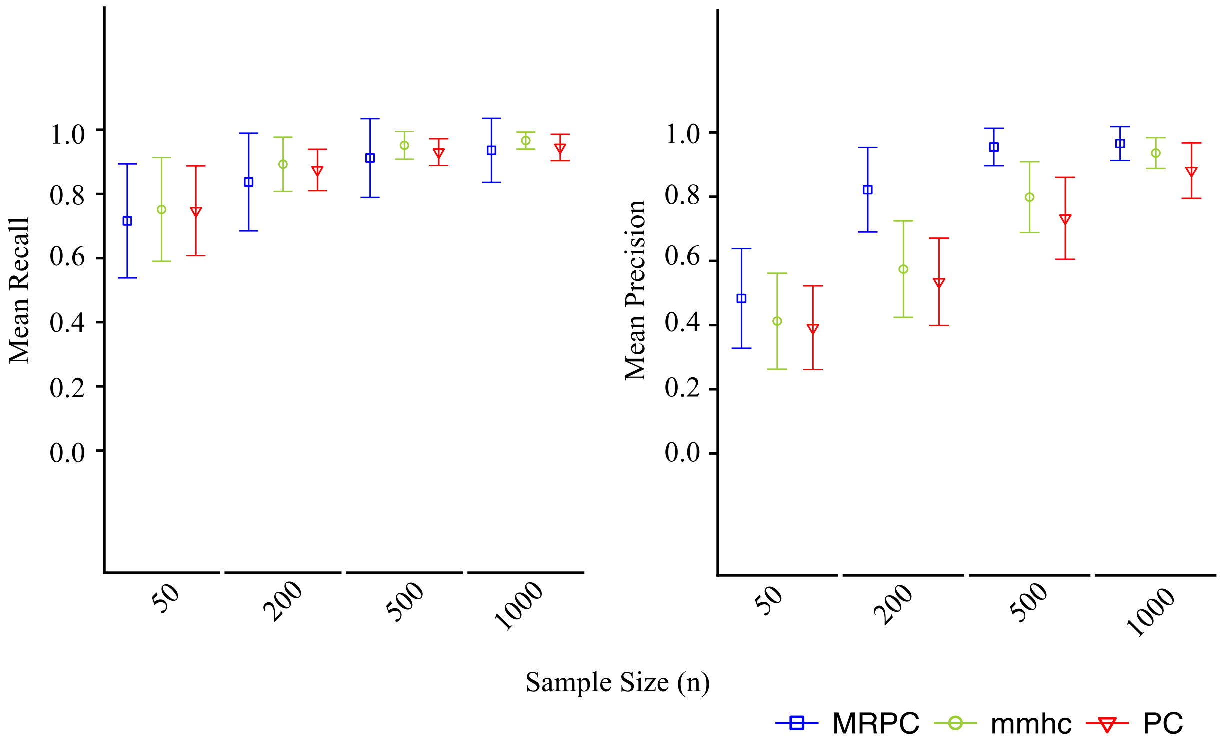

Figure S3: Mean and standard deviation of recall and precision of MRPC, mmhc and pc on data simulated under the complex topology with heterogeneous signal strengths. Similar to other simulations, 1000 data sets were generated under the complex topology. However, the signal strength (γ in the linear model) for any node was randomly chosen from 0.2, 0.5 and 1.0, with equal probability. We set FDR to be 0.05 when running MRPC, and the type I error rate to be 0.05 in mmhc and pc. Recall and precision were calculated as described in Section 2.6 in the main text.

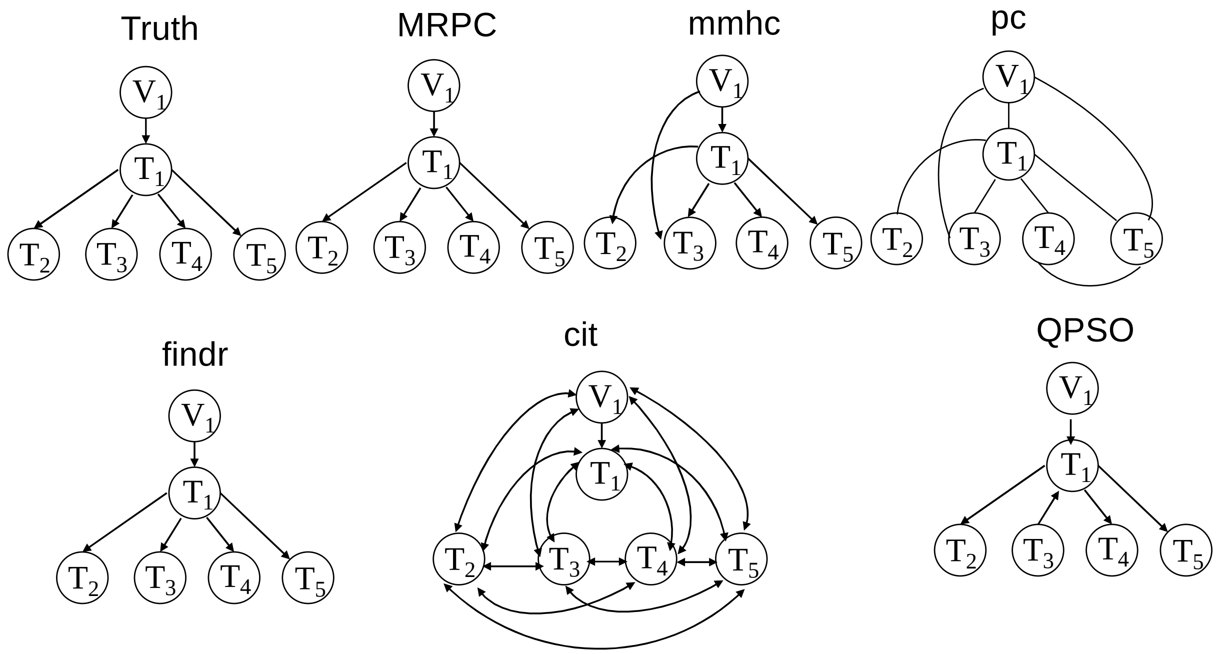

Figure S4: An example of inferred graphs from all six methods on data simulated under a star model with a large sample size (n =1000) and strong signal ($\gamma = 1.0$).

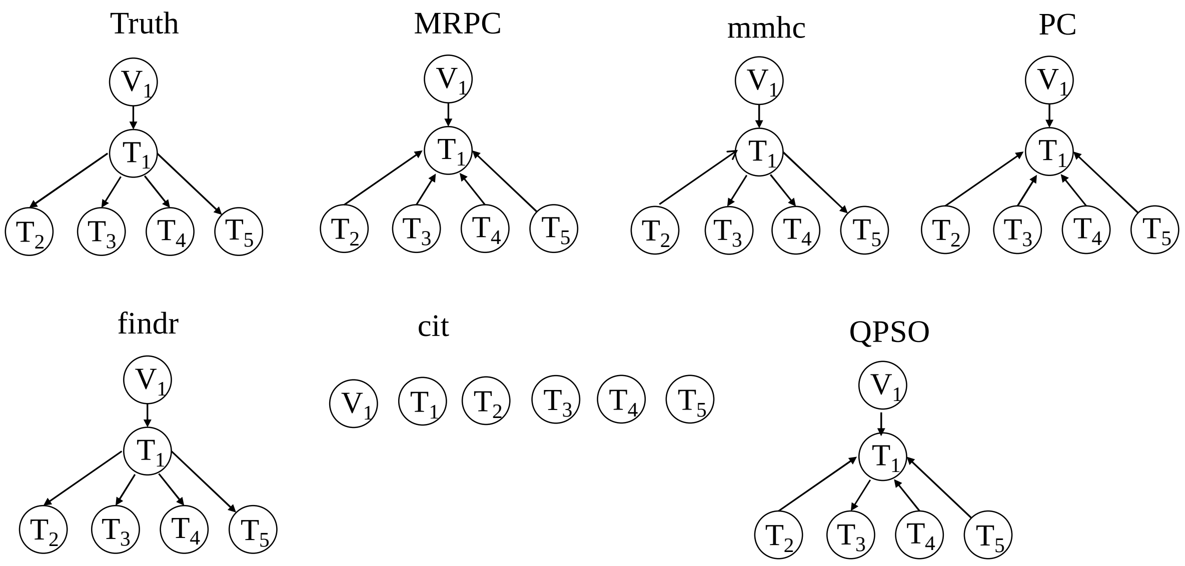

Figure S5: An example of inferred graphs from all six methods on data simulated under a star model with a large sample size (n =1000) and weak signal ($\gamma = 0.2$).

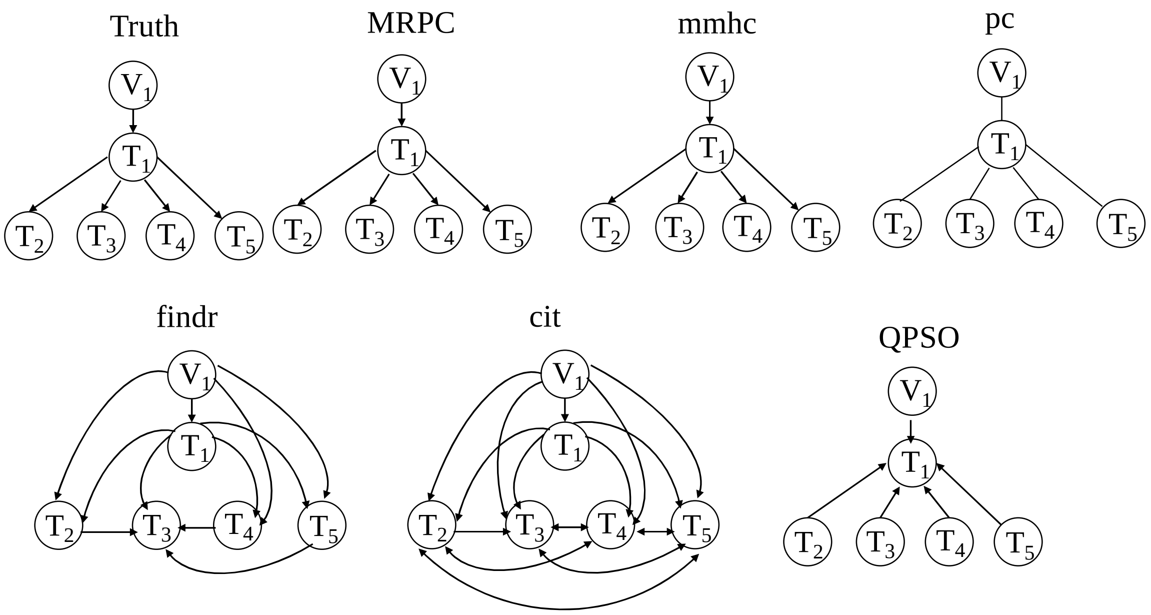

Figure S6: An example of inferred graphs from all six methods on data simulated under a star model with a large sample size (n =1000) and moderate signal ($\gamma = 0.5$).

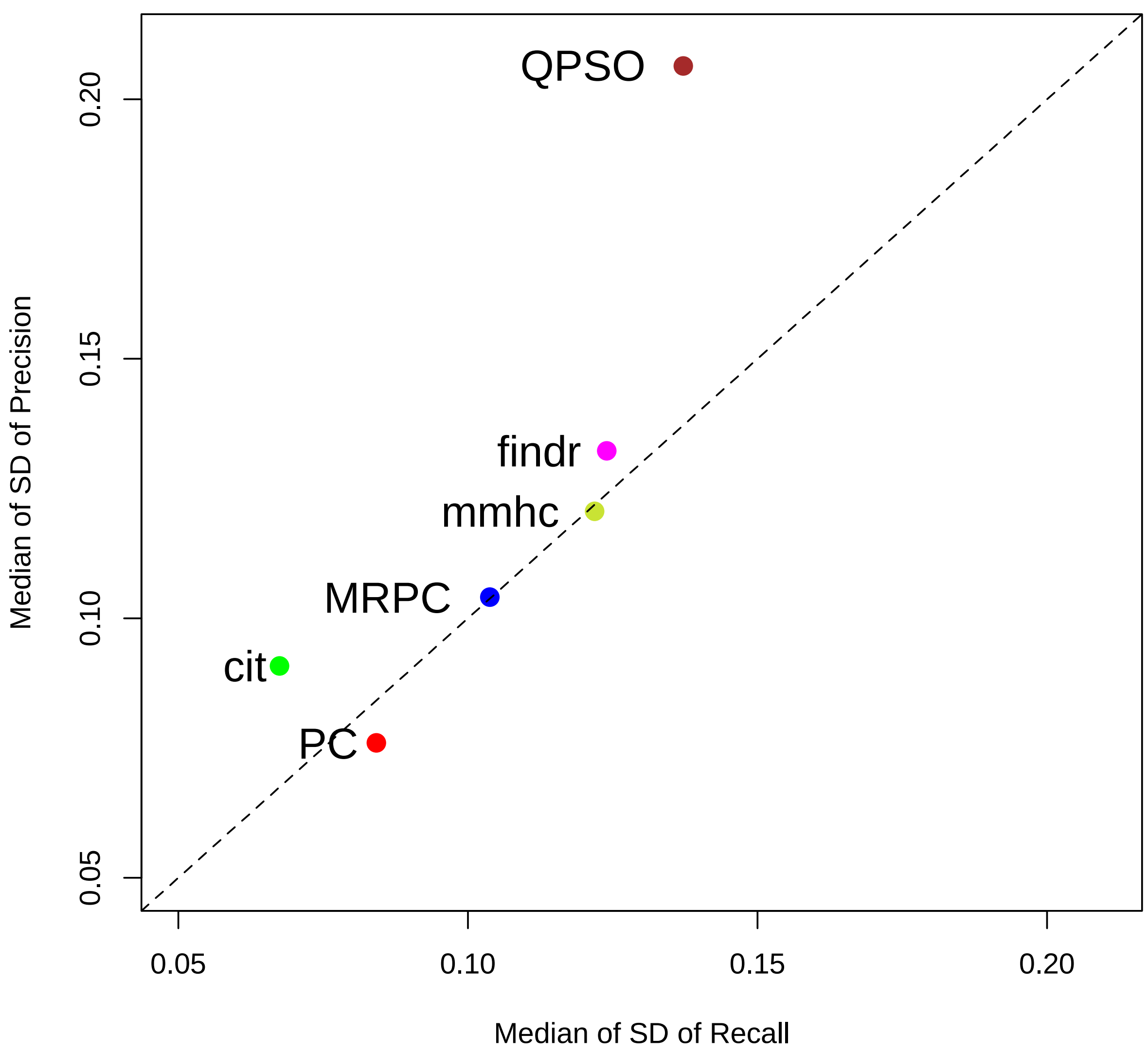

Figure S7: Median standard deviation of recall and precision of each method on simulated data across all parameter settings. Note that QPSO was run on only 20 (instead of 1000) data sets for each topology due to long runtime.

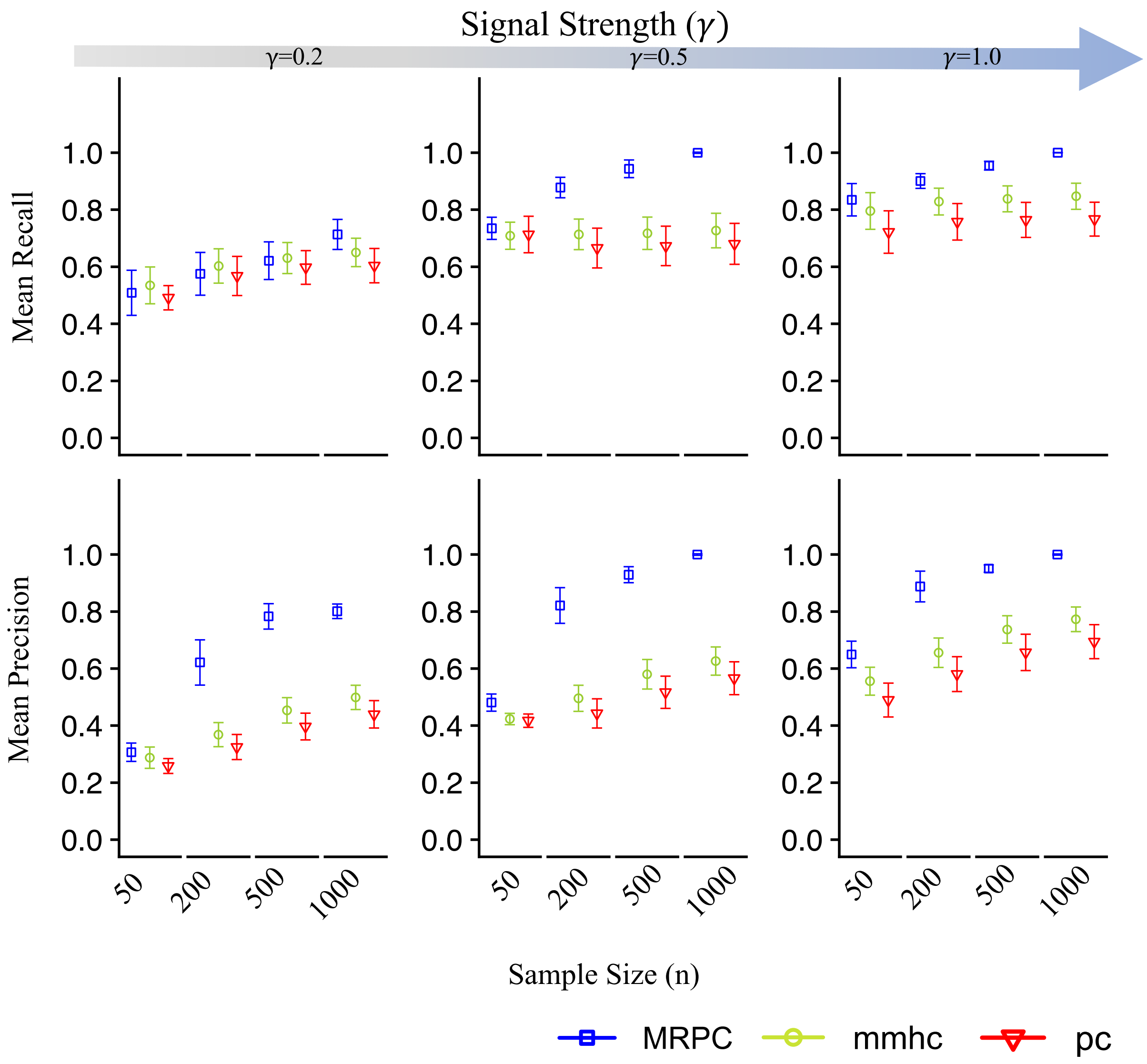

Figure S8: **Comparison of MRPC with mmhc from bnlearn and pc from pcalg with outliers present in the data.** Like **Figure S3,** we plot the mean recall and precision (with standard deviation) between the inferred graph and the simulation truth over all the simulated datasets. Only the complex topology in **Figure 3A** is used here. In each dataset, the majority of the genotype and molecular phenotype data were first simulated using the procedure described in Section 2.5 in the main text. Then 10 outliers were simulated from uniform distributions and mixed with the data. For each combination of parameters, we generated 1000 datasets with outliers and applied the three algorithms. We applied MRPC with robust correlation ($\beta = 0.005$) and FDR set to 0.05, as well as mmhc and pc with the single-test type I error rate set to 0.05.

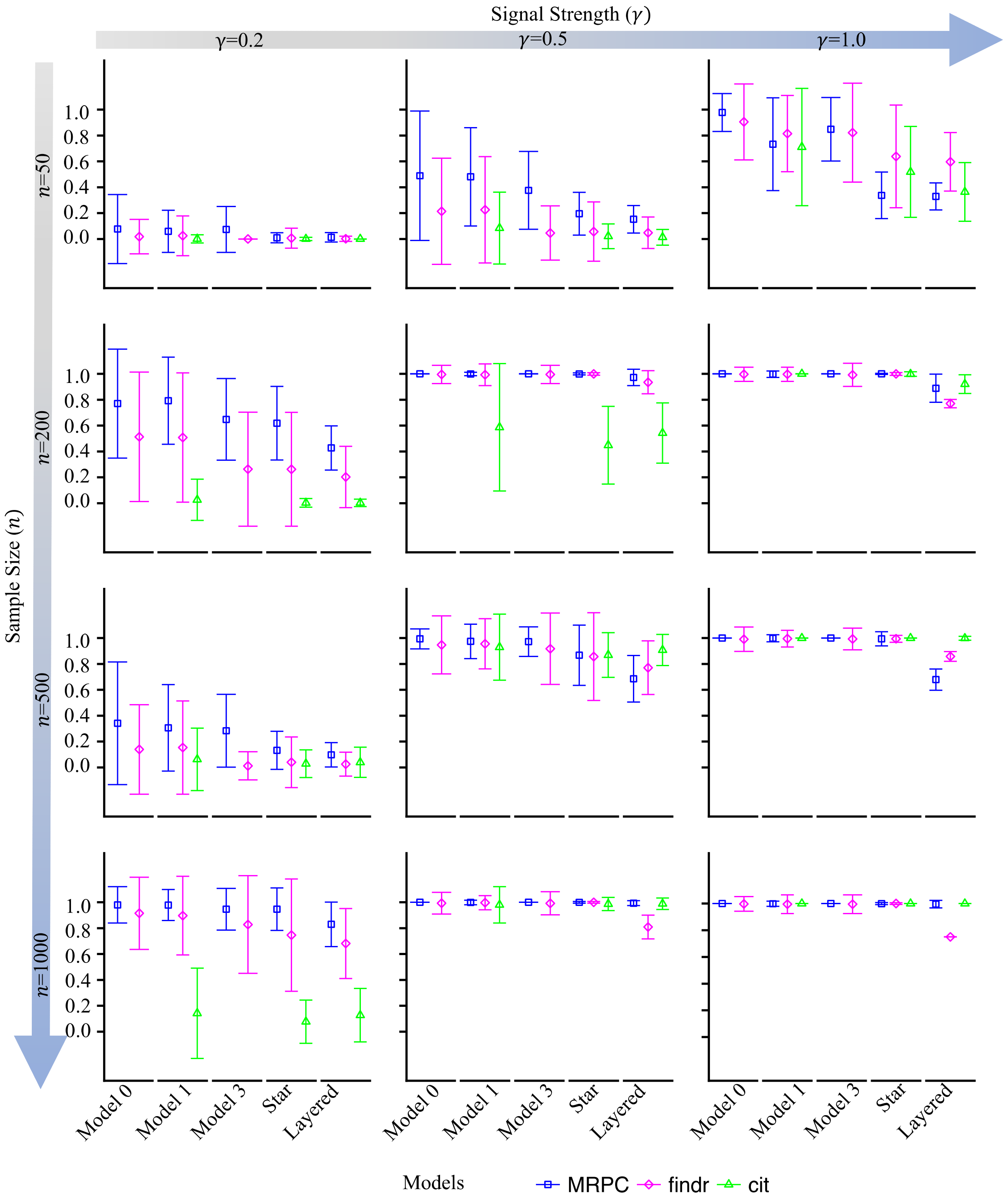

Figure S9: Recall for MRPC and two other PMR-based methods, findr and cit, when the graph skeleton is known to findr and cit. Since findr and cit cannot be applied to M2 or M4, these two models were excluded. Additionally, cit cannot be applied to M0 or M3, either. Therefore, the green bar representing cit is absent for M0 and M3. To include as many topologies as possible in this comparison, we calculated recall using all the edges in the true and inferred graphs. In other words, we did not exclude edges involving the genetic variant.

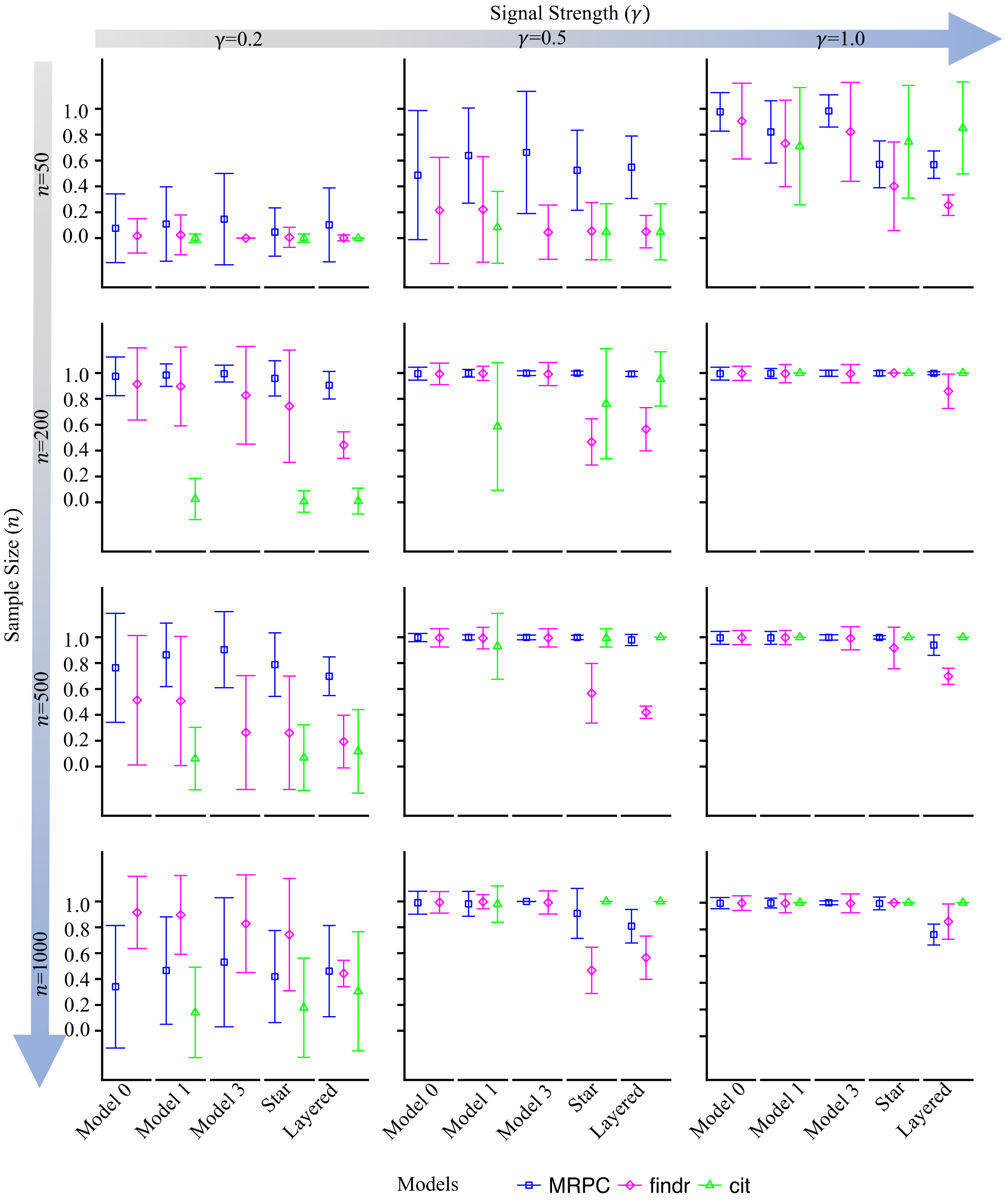

Figure S10: Precision for MRPC and two other PMR-based methods, findr and cit, when the graph skeleton is known to findr and cit. Since findr and cit can cannot be applied to M2 or M4, these two models were excluded. Additionally, cit cannot be applied to M0 or M3, either. Therefore, the green bar representing cit is absent for M0 and M3. To include as many topologies as possible in this comparison, we calculated precision using all the edges in the true and inferred graphs. In other words, we did not exclude edges involving the genetic variant.

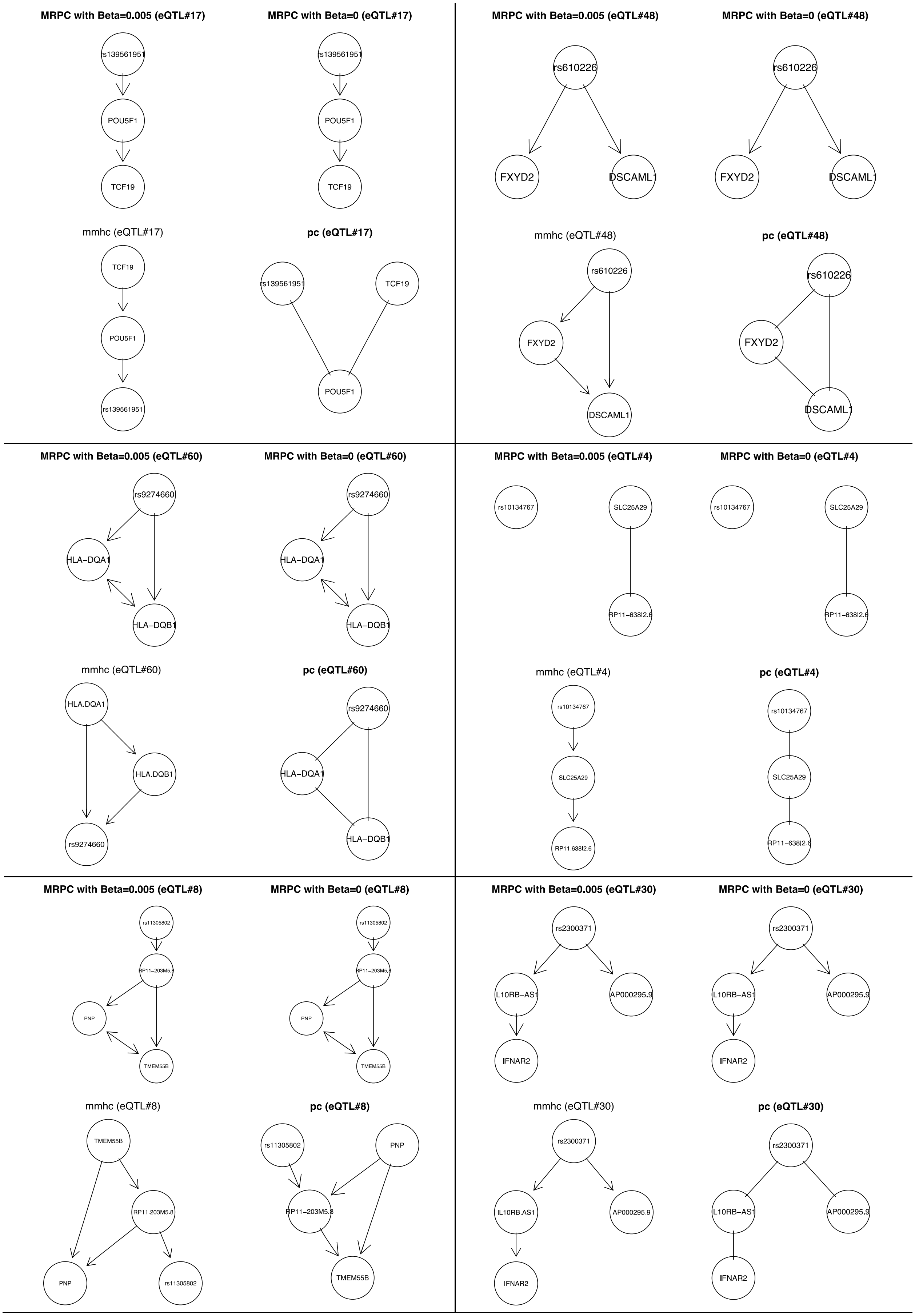
MRPC with Beta=0.005 (eQTL#17)
rs139561951
POU5F1
TCF19
MRPC with Beta=0 (eQTL#17)
rs139561951
POU5F1
TCF19
mmhc (eQTL#17)
TCF19
POU5F1
rs139561951
pc (eQTL#17)
rs139561951
TCF19
POU5F1
MRPC with Beta=0.005 (eQTL#48)
rs610226
FXYD2
DSCAML1
MRPC with Beta=0 (eQTL#48)
rs610226
FXYD2
DSCAML1
mmhc (eQTL#48)
rs610226
FXYD2
DSCAML1
pc (eQTL#48)
rs610226
FXYD2
DSCAML1
MRPC with Beta=0.005 (eQTL#60)
rs9274660
HLA−DQA1
HLA−DQB1
MRPC with Beta=0 (eQTL#60)
rs9274660
HLA−DQA1
HLA−DQB1
mmhc (eQTL#60)
HLA.DQA1
HLA.DQB1
rs9274660
pc (eQTL#60)
rs9274660
HLA−DQA1
HLA−DQB1
MRPC with Beta=0.005 (eQTL#4)
rs10134767
SLC25A29
RP11−638I2.6
MRPC with Beta=0 (eQTL#4)
rs10134767
SLC25A29
RP11−638I2.6
mmhc (eQTL#4)
rs10134767
SLC25A29
RP11.638I2.6
pc (eQTL#4)
rs10134767
SLC25A29
RP11−638I2.6
MRPC with Beta=0.005 (eQTL#8)
rs11305802
RP11−203M5.8
PNP
TMEM55B
MRPC with Beta=0 (eQTL#8)
rs11305802
RP11−203M5.8
PNP
TMEM55B
mmhc (eQTL#8)
TMEM55B
RP11.203M5.8
PNP
rs11305802
pc (eQTL#8)
rs11305802
PNP
RP11−203M5.8
TMEM55B
MRPC with Beta=0.005 (eQTL#30)
rs2300371
L10RB−AS1
AP000295.9
IFNAR2
MRPC with Beta=0 (eQTL#30)
rs2300371
L10RB−AS1
AP000295.9
IFNAR2
mmhc (eQTL#30)
rs2300371
IL10RB.AS1
AP000295.9
IFNAR2
pc (eQTL#30)
rs2300371
L10RB−AS1
AP000295.9
IFNAR2

Figure S11: Examples of the inferred causal graphs by different methods for eQTLs with multiple associated genes in the GEUVADIS data. Methods include MRPC with robust correlation (β=0.005), MRPC with Pearson correlation (β=0), mmhc (type I error rate=0.05) and pc (type I error rate=0.05). The European sample of 373 individuals were used here. In these examples, using the robust correlation and Pearson correlation leads to the same results in MRPC. mmhc sometimes wrongly infers the edge directions; e.g., for eQTL #17 in the top left panel, mmhc identifies an edge going from the gene to the SNP. pc generally does not infer the edge direction, as it does not implement the PMR and therefore treats the genotype nodes the same as the phenotype nodes.

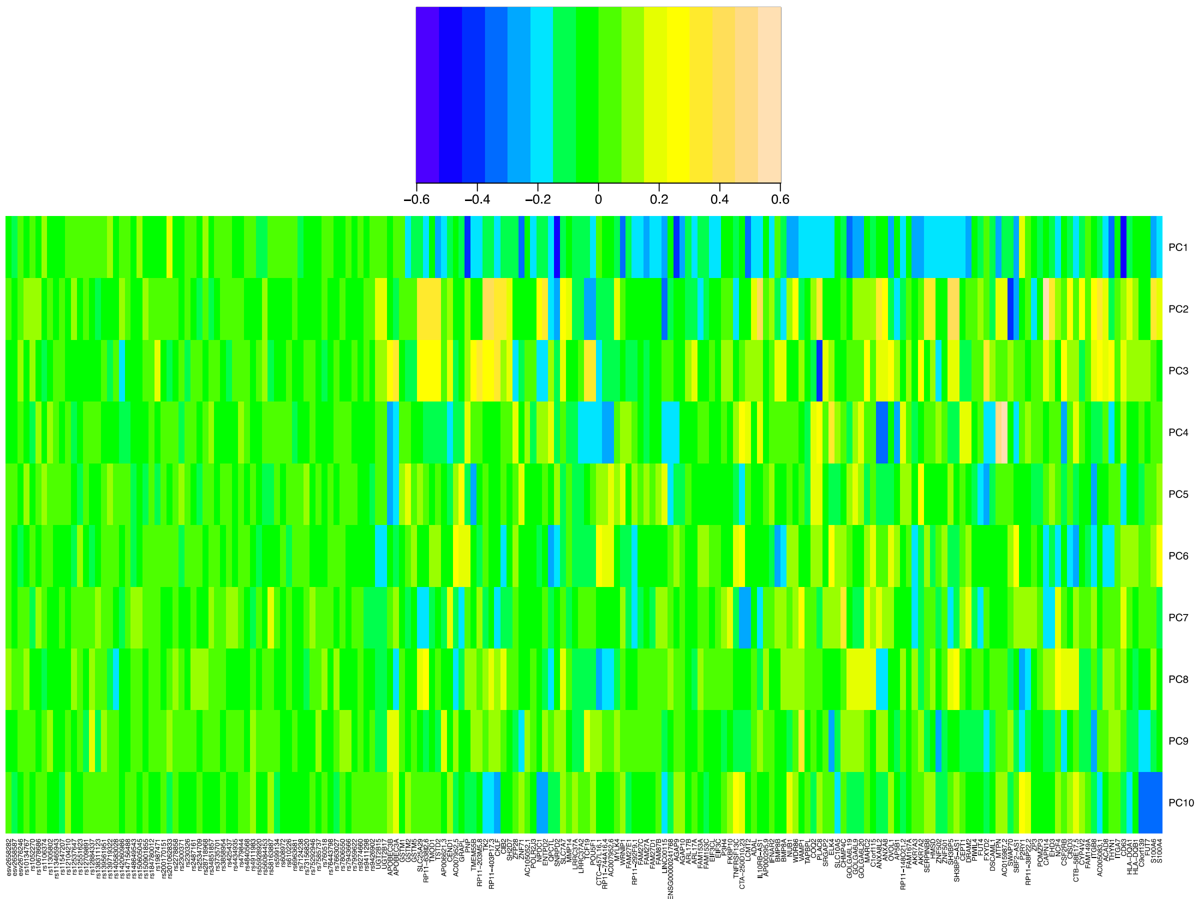

Figure S12: Correlation heatmap of eQTLs, genes and principal components (PCs) from GEUVADIS. The eQTLs and genes are those from the 62 eQTL-gene sets. PCs are obtained from performing the PCA on the entire gene expression data (see details in Section 3.4 in the main text).

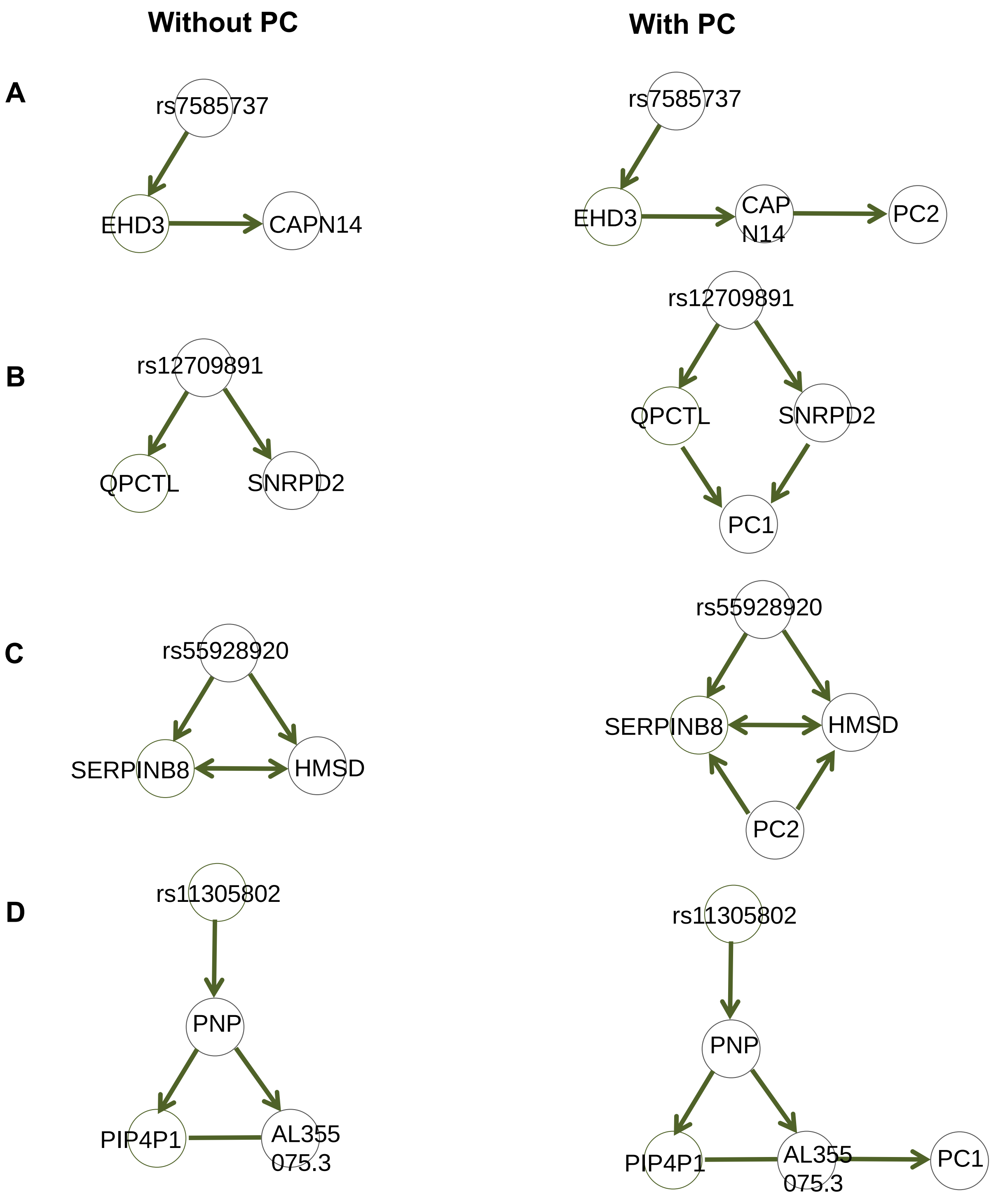

Figure S13: Examples of networks inferred by MRPC for certain eQTL-gene sets with and without the principal components (PCs). PCs were inferred from the entire gene expression data, which are therefore linear combinations of genes typically not in an eQTL-gene set. We did not observe any case in which inclusion of PCs in the MRPC analysis altered the network inferred for the eQTL and genes.
